# Impairment of autophagy after spinal cord injury potentiates neuroinflammation and motor function deficit in mice

**DOI:** 10.7150/thno.72713

**Published:** 2022-07-11

**Authors:** Yun Li, Zhuofan Lei, Rodney M. Ritzel, Junyun He, Hui Li, Harry M. C. Choi, Marta M. Lipinski, Junfang Wu

**Affiliations:** Department of Anesthesiology and Center for Shock, Trauma and Anesthesiology Research (STAR), University of Maryland School of Medicine, Baltimore, MD, 21201 USA.

**Keywords:** Autophagy, Beclin-1, Spinal cord injury, Neuroinflammation, Microglia

## Abstract

Autophagy is a catabolic process that degrades cytoplasmic constituents and organelles in the lysosome, thus serving an important role in cellular homeostasis and protection against insults. We previously reported that defects in autophagy contribute to neuronal cell damage in traumatic spinal cord injury (SCI). Recent data from other inflammatory models implicate autophagy in regulation of immune and inflammatory responses, with low levels of autophagic flux associated with pro-inflammatory phenotypes. In the present study, we examined the effects of genetically or pharmacologically manipulating autophagy on posttraumatic neuroinflammation and motor function after SCI in mice.

**Methods:** Young adult male C57BL/6, CX3CR1-GFP, autophagy hypomorph *Becn1^+/-^* mice, and their wildtype (WT) littermates were subjected to moderate thoracic spinal cord contusion. Neuroinflammation and autophagic flux in the injured spinal cord were assessed using flow cytometry, immunohistochemistry, and NanoString gene expression analysis. Motor function was evaluated with the Basso Mouse Scale and horizontal ladder test. Lesion volume and spared white matter were evaluated by unbiased stereology. To stimulate autophagy, disaccharide trehalose, or sucrose control, was administered in the drinking water immediately after injury and for up to 6 weeks after SCI.

**Results:** Flow cytometry demonstrated dysregulation of autophagic function in both microglia and infiltrating myeloid cells from the injured spinal cord at 3 days post-injury. Transgenic CX3CR1-GFP mice revealed increased autophagosome formation and inhibition of autophagic flux specifically in activated microglia/macrophages. NanoString analysis using the neuroinflammation panel demonstrated increased expression of proinflammatory genes and decreased expression of genes related to neuroprotection in *Becn1^+/-^* mice as compared to WT controls at 3 days post-SCI. These findings were further validated by qPCR, wherein we observed significantly higher expression of proinflammatory cytokines. Western blot analysis confirmed higher protein expression of the microglia/macrophage marker IBA-1, inflammasome marker, NLRP3, and innate immune response markers cGAS and STING in *Becn1^+/-^* mice at 3 day after SCI. Flow cytometry demonstrated that autophagy deficit did not affect either microglial or myeloid counts at 3 days post-injury, instead resulting in increased microglial production of proinflammatory cytokines. Finally, locomotor function showed significantly worse impairments in *Becn1^+/-^* mice up to 6 weeks after SCI, which was accompanied by worsening tissue damage. Conversely, treatment with a naturally occurring autophagy inducer trehalose, reduced protein levels of p62, an adaptor protein targeting cargo to autophagosomes as well as the NLRP3, STING, and IBA-1 at 3 days post-injury. Six weeks of trehalose treatment after SCI led to improved motor function recovery as compared to control group, which was accompanied by reduced tissue damage.

**Conclusions:** Our data indicate that inhibition of autophagy after SCI potentiates pro-inflammatory activation in microglia and is associated with worse functional outcomes. Conversely, increasing autophagy with trehalose, decreased inflammation and improved outcomes. These findings highlight the importance of autophagy in spinal cord microglia and its role in secondary injury after SCI.

## Introduction

Autophagy is an essential catabolic process for maintaining healthy and stable homeostasis in the central nervous system (CNS). Accumulation of autophagosomes has been detected after traumatic spinal cord injury (SCI) 1, 2, but its mechanisms and function remain unknown. We recently demonstrated that autophagy flux - the progress of substrates through autophagic compartments leading to their delivery and degradation in the lysosomes - is blocked after SCI in mouse and rat models 3, 4. In neurons this is due to a temporary decrease in lysosomal function leading to pathological accumulation of dysfunctional autophagosomes 4, 5. Prior studies 6, 7 including ours 3-5 have reported that defects in autophagy-lysosomal function contribute to neuronal damage and neuronal cell death after SCI. Recent data indicate that perturbation of autophagy can also affect inflammatory responses, with high levels of autophagy flux associated with anti-inflammatory, and low levels with pro-inflammatory phenotypes 8, 9. Prior studies have reported on the potential role of autophagy in microglial activation and its subsequent effects on neuroinflammation in other disease models 10-13. However, direct evidence that links autophagy to modulation of neuroinflammation after SCI is limited and the results are conflicting.

The initial traumatic event of contusion SCI triggers a signaling cascade leading to glial activation, neuroinflammation, and neuronal cell death. These delayed and progressively worsening secondary injury processes contribute to tissue loss and neurological dysfunction. Neuroinflammation within the injury site is caused by both activated microglia and peripheral myeloid cells infiltrating the spinal cord. These microglia/macrophages can either play a restorative or destructive role, depending upon the degree of injury severity and expression level of neuroprotective versus neurotoxic factors. Recent studies demonstrated that restoration of autophagy flux in microglia/macrophages can alleviate neuroinflammation and exert neuroprotective effects in Alzheimer's disease and ischemic brain 14, 15. Based on this evidence, we hypothesized that autophagy deficiency would polarize microglia/macrophages towards a more pro-inflammatory activation state after SCI. Beclin-1 (BECN1) is a core subunit of the phosphatidylinositol-3 kinase (PI3K) complex necessary for autophagosome formation 16. Beclin-1 acetylation inhibits autophagosome maturation, leading to impairment in autophagic flux 17. In cultured hippocampal HT22 neurons, Fekadu and Rami 18 reported that Beclin-1 deficiency impaired the formation of autophagosomes and lysosome biogenesis leading to neuronal cell death. Additional evidence showed that Beclin-1 is required for the fusion of lysosomes to autophagosomes 19. As Beclin-1 is an autophagy gene essential for early embryonic development, Beclin-1 knockout mice (*Becn^-/-^*) die early in embryogenesis and Beclin-1 heterozygous (*Becn1^+/-^*) mice display defect in induction of autophagy 20. Our study first examined the effect of SCI on autophagy in microglia/macrophages and then used *Becn1^+/-^* mice to explore posttraumatic neuroinflammation and motor function in the context of inhibited autophagic flux.

Numerous studies throughout the years sought to promote functional recovery after SCI by enhancing autophagy via pharmacological methods 1, 21. Most notable of these is rapamycin, a drug that enhances autophagy by inhibiting mammalian target of rapamycin (mTOR) 6, 22-24. However, the mTOR pathway is also a crucial for protein synthesis, axonal growth, and immune responses 25-27. As a potential alternative, mTOR-independent inducers of autophagy, such as the disaccharide trehalose, have been utilized to promote neuroprotection in animal models of neurodegenerative disease and in a rabbit model of spinal cord ischemia 28-30. In the present study, we investigated the effects of enhancing autophagy by trehalose on neuroinflammation and motor function after SCI in mice.

We observed accumulation of autophagosomes and inhibition of autophagy flux specifically in the activated microglia/macrophages in the injured spinal cord. After SCI, autophagy hypomorph *Becn1^+/-^* mice displayed potentiated neuroinflammation and increased production of proinflammatory cytokines in microglia, resulting in worse functional recovery and increased tissue damage. Trehalose treatment reestablished autophagy flux, accompanied by reduced neuroinflammation and better recovery following SCI. Together, these findings strongly implicate autophagy in modulation of neuroinflammation after SCI and support therapeutic potential of promoting autophagy as a treatment after injury.

## Methods

### Mouse spinal cord injury contusion model and drug treatment

Young adult male or female (10-12 weeks, 20-25 g) C57BL/6 and CX3CR1-GFP mice (B6.129P-Cx3cr1tm1Litt/J, Cat# 005582) were obtained from The Jackson Laboratory. Beclin1 heterozygous (*Becn1^+/-^*) mice purchased from The Jackson Laboratory were bred in the animal facility. Mice were housed on a 12 hours (h) light/dark cycle with food and water provided for them *ad libitum*. According to the 2019 SCI Data Sheet from the National SCI Statistical Center, SCI is more common in men (~80%). Thus, male mice were used in the present study. Moderate spinal cord contusion injury was conducted using the Infinite Horizon (Precision Systems and Instrumentation) spinal cord impactor as previously described 5, 31. Briefly, after anesthetization with isoflurane, the spinal cord at the T10 level was exposed with a laminectomy and the spinal column was stabilized using the steel clamps over the bilateral processes at T9 and T11. A midline spinal contusion at T10 level with a force of 60-65 kilodyne (kdyn) was conducted. The bladders of injured mice were manually voided 2-3 times daily until a reflex bladder was established. Animals in the sham surgery group underwent the same procedure as SCI mice except for the laminectomy and contusion impact. All animal experiments and surgical procedures were performed according to protocols approved by the Institutional Animal Care and Use Committee (IACUC) at the University of Maryland, School of Medicine.

In order to assess whether enhancement of autophagy could mitigate neuroinflammation and functional deficits after SCI, young adult male C57BL/6 mice were fed trehalose, a sugar analog and an mTOR independent autophagy inducer, or sucrose control starting on the date of injury until the time of sacrifice. Based on results from prior studies 32-34 and our pilot data, at 10 min post-injury, mice were given 5% trehalose (Cat# T9449, Sigma-Aldrich) or 5% sucrose (Cat# S9378, Sigma-Aldrich) via oral gavage twice per day up to 3 days in addition to treated drinking water. For chronic studies, mice were administrated 5% trehalose or sucrose via oral gavage twice per day for the first week followed by continuous administration at 2.5% trehalose or sucrose in their drinking water for 6 w.

After surgery, all mice were assigned to one of four groups based on surgery (sham or SCI) and treatment (trehalose or sucrose control) according to a randomized block experimental design. The surgical procedures and all behavioral tests were carried out with the same equipment by blinded experimenters. The number of mice at various time points in each study is indicated in the figure legends.

### Motor function tests

Hindlimb locomotor function was assessed using the Basso mouse scale (BMS) and the BMS subscore as previously described 35, 36 on day 1 and 3 after injury, followed by weekly assessments thereafter for up to 6 w. In addition, horizontal ladder test was performed at 6 w post-injury to assess motor coordination for evaluating skilled walking for mice 37. Training at 1d prior to testing consisted of a 5 min acclimation period where the tested animal was placed on a 60 cm in length horizontal ladder apparatus and home cage followed by at least 5 trials of directing the animal to run across the ladder beam towards the home cage. In between trials, each mouse was allowed for resting on the border for 1 min for habituation. All test trials were videotaped with a Canon camcorder and each animal was filmed for a minimum of 5 eligible trials. A good and eligible trial consisted of a full view of hindlimbs while the mouse crossed the horizontal beam at consistent rate across all 20 rungs without turning around. The stepping for each rung was assessed by the rater for three types of positive events: Plantar step, Toe step, Skip; and three types of negative events: Slip, Miss, Drag. Final data of the horizontal ladder test was presented as a ladder beam score (LBS %), which is the percentage number of positive events in total number of events and the cumulative number of errors/negative events across five trails.

### Flow cytometry

Mice were perfused with 40 mL of cold PBS in a staggered order between groups and the fresh ~1 cm of spinal cord tissue surrounding the epicenter of the lesion site was weighed to control for any variation and normalize cell counts. The elapsed time between the first and last mouse was approximately 60-90 minutes. Tissue was placed separately in complete Roswell Park Memorial Institute (RPMI) 1640 (Cat# 22400105, Invitrogen) medium and mechanically and enzymatically digested in collagenase/dispase (Cat# 10269638001, 1 mg/mL; Roche Diagnostics), papain (Cat# LS003119, 5 U/mL; Worthington Biochemical), 0.5 M EDTA (Cat# 15575020, 1:1000; Invitrogen), and DNAse I (Cat# 10104159001, 10 mg/mL; Roche Diagnostics) for 1 h at 37 °C on a shaking incubator (200 rpm). The cell suspension was washed twice with RPMI, filtered through a 70 μm filter, and RPMI was added to a final volume of 5 mL/spinal cord segment and kept on ice. Cells were then transferred into FACS tubes and washed with FACS buffer. Cells were then incubated with Fc Block (Cat# 101320, Clone: 93; Biolegend) for 10 min on ice and stained for the following surface antigens: CD45-eF450 (Cat# 48-0451-82, Clone: 30-F11; eBioscience), CD11b-APC/Fire™750 (Cat# 101262, Clone: M1/70; Biolegend), Ly6C-APC (Cat# 128016, Clone: HK1.4; Biolegend), Ly6G-PE (Cat# 127607, Clone: 1A8; Biolegend), and Zombie Aqua fixable viability dye (Cat# 423102, Biolegend). Cells were then washed in FACS buffer, fixed in 2% paraformaldehyde for 10 min, and washed once more prior to adding 500 μL FACS buffer. Intracellular staining for LAMP1-PerCPCy5.5 (Cat# 121626, Clone: 1D4B; Biolegend), LAMP2-PE (Cat# 108506, Clone: H4B4; Biolegend), and P62/sqstm1-AF647 (Cat# 42822AF647, Novus Biologicals) was performed after fixation/permeabilization. Intracellular cytokine staining for IL1β-PerCP-eF710 (Cat# 46-7114-82, Clone: NJTEN3; Invitrogen) and TNF-PE/Cy7 (Cat# 506324, Clone: MP6-XT22; Biolegend) was performed after 2 h incubation with brefeldin A at 37 °C followed by fixation/permeabilization as previously described 31, 38, 39. Cyto-ID Autophagy Detection Kit (Cat# ENZ-51031-K200, Enzo Life Sciences) and LysoTracker probe (Cat# L7526, Invitrogen) were used according to the manufacturer's instructions.

Data were acquired on a BD LSRFortessa cytometer using FACSDiva 6.0 (BD Biosciences) and analyzed using FlowJo (Treestar Inc.). Countbright™ Absolute Counting Beads (Invitrogen) were used to estimate cell counts per the manufacturer's instructions. Data were expressed as either cells/mg tissue weight. Leukocytes were first gated using a splenocyte reference (SSC-A vs FSC-A). Singlets were gated (FSC-H vs FSC-W), and live cells were gated based on Zombie Aqua exclusion (SSC-A vs Zombie Aqua-Bv510). Resident microglia were identified as the CD45^int^CD11b^+^Ly6C^-^ population, and CNS-infiltrating leukocytes were identified as CD45^hi^CD11b^+^ myeloid cells or CD45^hi^CD11b^-^ lymphocytes. Within the CD45^hi^ myeloid subset, monocytes were identified as Ly6C^hi^Ly6G^-^ and neutrophils, Ly6C^+^Ly6G^+^ 40, 41 (**Figure S1**).

### NanoString analysis

At the designed time point, mice were euthanized and perfused with 50 mL ice cold normal saline. Total RNA was extracted from the dissected 0.5-cm segment of spinal cord centered at the injury epicenter (lesion area) using a miRNeasy isolation kit (Cat# 217084, Qiagen). Total RNA (20 ng/μL) was run on an nCounter® Mouse Neuroinflammation v1.0 panel (NanoString Technologies, Seattle, WA) to simultaneously measure RNA transcript counts for 757 genes and 13 housekeeping genes 41. Sample gene transcript counts were normalized with NanoString's nSolver Analysis Software Version 4.0 that uses a geoNorm algorithm to identify stable housekeeping genes for normalization 42. For visualization of the data, we used the VennDiagram and EnhancedVolcano packages in R for plotting out venn diagrams and volcano plots. For pathway analysis, we used the integrative web-based application Enrichr for enrichment profiling of differentially expressed genes derived from pairwise comparison between groups 43-45.

### Real-time quantitative PCR (qPCR)

Complementary DNA (cDNA) was synthesized by a Verso cDNA RT kit (Cat# AB1453B, Thermo Scientific) per the manufacturer's protocol. Real-time PCR for target mRNAs was performed using TaqMan gene expression assays for Lipocalin 2 (Lcn2, Mm01324470_m1), Ccl2 (Mm00441242_m1), Ccl7 (Mm00443113_m1), Gbp2 (Mm00494576_g1), IL-1β (Mm0133-6189_m1), Myc (Mm00487804_m1), IL-6 (Mm00446190_m1), suppressor of cytokine signalling 3 (SOCS3, Mm00545913_s1), C5ar1 (Mm00500292_s1), Trem2 (Mm04209424_g1), Ptgs2 (Mm00478374_m1), Nlrp3 (Mm00840904_m1), Mb21d1 (Mm01147496_m1), Tmem173 (Mm00727224_s), Tnfrsf1 (Mm00441883_g1), Fos (Mm00487425_m1), Tgm2 (Mm00436979_m1), Lamp1 (Mm01217070_m1), Pink1 (Mm00550827_m1), Apoe (Mm01307192_m1), Blnk (Mm01197846_m1), and GAPDH (Mm99999915_g1) (Applied Biosystems, Carlsbad, CA] on an QuantStudio™ 5 Real-Time PCR System (Applied Biosystems). Gene expression was normalized by GAPDH and compared to the control sample to determine relative expression values by the 2-ΔΔCt method 31, 41.

### Tissue processing and histological analysis

Following intracardial perfusion with ice cold normal saline and 4% paraformaldehyde, mice spinal cord segments containing the lesion area were dissected out, embedded, and cut into 20-μm-thick serial sections placed serially on a set of 10 slides for 10 sets of slides. Luxol fast blue (LFB, Cat# S3382, Sigma-Aldrich) staining was performed to determine the location of the lesion epicenter, which is defined as the section with the least amount of spared white matter (SWM). Residual WM was also calculated for areas rostral and caudal to the lesion epicenter for assessment of SWM 34. Images were captured at ×2.5 magnification and analyzed with the National Institutes of Health ImageJ software (RRID:SCR_003070). The threshold level of each 8-bit image was set to mark only LFB-positive tissue, and total LFB-positive area was calculated for each section. For assessment of lesion volume, sections spaced 1 mm apart from 5 mm rostral to 5 mm caudal the injury epicenter were stained with GFAP (1:1000; Cat# Z0334, Dako) and DAB (Cat# PK-6100, Vector Labs) as the chromogen. Quantification was based on the Cavalieri method using Stereoinvestigator Software (MBF Biosciences), as previously described 31, 41.

### Immunohistochemistry (IHC) and quantitative image analysis

Coronal spinal cord sections from CX3CR1-GFP mice at 3 d after SCI were applied for IHC staining followed procedures described previously 36, 41. The following primary and secondary antibodies were used: p62/SQSTM1 (1:500; Cat# GP62-C-WBC, Progen), LC3B (1:400; Cat# NB100-2220, Novus Biologicals), rat anti-F4/80 (1:600, Cat# ab6640, Abcam), Alexa FluorTM 546 goat anti-rabbit IgG (1:800, Cat# A11035, Invitrogen), and Alexa FluorTM 647 goat anti-rat IgG (1:800, Cat# A21247, Invitrogen). The green channel was left blank for imaging of CX3CR1-GFP. All the images were acquired using a Nikon A1 Laser Confocal microscope. Representative CX3CR1-GFP/p62/F4/80 images were obtained by tile scan. Five sections per mouse around the lesion core with a 200 μm interval were selected for imaging. For each section, the dorsal white matter region was imaged by 6 x 4 tile scan with identical imaging parameters under a 60x lens. In the NIH ImageJ software (1.53), every single region, consisting of 5 layers to cover the entire depth, was processed by the Z-axis projection for quantification. The number of GFP+, p62+, LC3+, and F4/80+ cells were quantified manually in ImageJ after background subtraction and signal filtering. The average values of all 5 sections were taken into next statistical analysis. All IHC staining experiments were performed with appropriate positive control tissue, as well as primary/secondary only negative controls.

In addition, primary antibodies PhosphoDetect™ Anti-Neurofilament-H Mouse mAb (SMI-312, 1:1000; Sigma-Aldrich, NE1022) and NeuN (1:500; Millipore, MAB377) were applied for IHC staining in chronic SCI tissues. Images were acquired 0.5-1.5mm rostral to the epicenter, with n = 5 images/5 sections per mouse. All images were captured using a fluorescent Nikon Ti-E inverted microscope, at 20X (CFI Plan APO VC 20X NA 0.75 WD 1 mm) magnification and the background of each image was subtracted using background ROI. The number of NeuN+ cells in the grey matter and SMI312+ intensity in the whiter matter were quantified using the NIH Image J software (1.43; NIH).

### Sample preparation and western blot analysis

Approximately 5 mm of spinal cord tissue was excised from the lesion area of each mouse sacrificed at indicated time-points. For sham animals, an equal length of 5 mm was extracted from the approximate area of T10. Western blot analysis was performed as previously described 5, 31. Briefly, tissue samples were sonicated in RIPA lysis buffer (Cat# R0278, Sigma-Aldrich) supplemented with 1 x protease inhibitor cocktail (Cat# P8340, Sigma-Aldrich), Phosphatase Inhibitor Cocktail 2 (Cat# P5726, Sigma-Aldrich) and Phosphatase Inhibitor Cocktail 3 (Cat# P0044, Sigma-Aldrich), followed by centrifugation at 20,000 x g for 20 min. Protein concentration was determined by the pierce BCA method (Cat# 23227, Thermo-Fisher Scientific). Samples were run on 4-20% SDS-PAGE (Bio-Rad, US) and transferred to 0.2 μm nitrocellulose membrane (Bio-Rad). Membranes were blocked with 10% non-fat skim milk in PBST, incubated overnight with primary antibodies diluted in blocking buffer and incubated for 2 h in HRP-conjugated secondary antibodies. After the immunoblots were visualized with SuperSignal West Dura Extended Duration Substrate (Cat# 34076, Thermo-Fisher Scientific) and imaged with ChemiDoc TM MP system (Bio-Rad), the optical density of signal bands was quantified by Image Lab software (Bio-Rad). Primary antibodies and respective dilution rates are as followed: LC3 (1:4000; Cat# NB100-2220, Novus Biologicals), p62/SQSTM1 (1:1000; Cat# 610832, BD Bioscience), BECLIN-1 (1:500; Cat# 3495S, Cell Signaling Technologies), LAMP1 (1:200; Cat# 1D4B, Developmental Studies Hybridoma Bank), NLRP3 (1:500; Cat# 15101S, Cell Signaling Technologies), cGAS (1:1000; Cat# 31659S, Cell Signaling Technologies), STING (1:1000, Cat# 13647S, Cell Signaling Technologies), rabbit anti-Ionized calcium binding adaptor molecule 1 (Iba-1, 1:1000, Cat# 019-19741, Wako Chemicals), and β-actin (1:10,000; Cat# SAB1305567A1978, Sigma-Aldrich). The data presented reflects the intensity of a target protein band normalized by actin and compared to Sham/WT for each sample (expressed in the fold of Sham/WT).

### Statistical analysis

Data are presented as mean ± SEM, and individual data points are shown for each graph. Statistical analysis was performed using the GraphPad Prism Software, version 8.3.0 for Windows (GraphPad Software; RRID: SCR_002798) or SigmaPlot, version 12 (Systat Software; RRID: SCR_003210). BMS scores and the subscore were analyzed using two-way ANOVA with repeated measures followed by Holm-Sidak's post hoc test for multiple comparisons. For multiple comparisons, one-way or two-way ANOVA were performed followed by Tukey's post hoc test for multiple comparisons of parametric (normality and equal variance passed) data. Nonparametric Mann-Whitney U tests were applied to the data that did not pass normality test. Stereological data for lesion volume and the cell counts for IHC were analyzed using Student's t test. Statistical analysis of each assay is detailed in each figure legend. A p value of < 0.05 was considered statistically significant.

Statistical analysis of NanoString data was performed in RStudio Version 1.2.5033. Multidimensional scaling (MDS) was performed with the “cmdscale()”function, in which distance between each sample was calculated with the log2 method. Differential expression (DE) analysis between paired groups were performed with the nSolver Analysis Software (Version 4.0, NanoString Technologies Inc.), which performs normalization with raw gene transcript counts, positive and negative controls, and housekeeping gene transcript data provided by NanoString as described in the user manual 46. The four pairwise comparisons were described in the current study as follows: (1) Sham/*Becn1^+/-^* vs. Sham/WT -Comparison 1; (2) SCI/WT vs. Sham/WT -Comparison 2; (3) SCI/* Becn1^+/-^* vs. Sham/*Becn1^+/-^* - Comparison 3; and (4) SCI/* Becn1^+/-^* vs. SCI/WT - Comparison 4. All comparisons “Group 1 vs. Group 2” were interpreted as “Group 1 relative to Group 2” in the text and figures. During analysis, we utilized the Benjamini-Hochberg method for false discovery rate (FDR) correction, and a non-adjusted p-value less than 0.05 was used to identify differentially expressed (DE) genes in each comparison. Subsets of DE genes were normalized across samples as z-scores and then averaged to a single value per group before being plotted in GraphPad Prism 8.3.0 and displayed as heatmaps. Pathway enrichment analysis was performed with the Enrichr application using the Molecular Signatures Database (MSigDB) Hallmark 2020.

## Results

### SCI impairs autophagy flux in microglia and infiltrating monocytes

**Figure 1:**
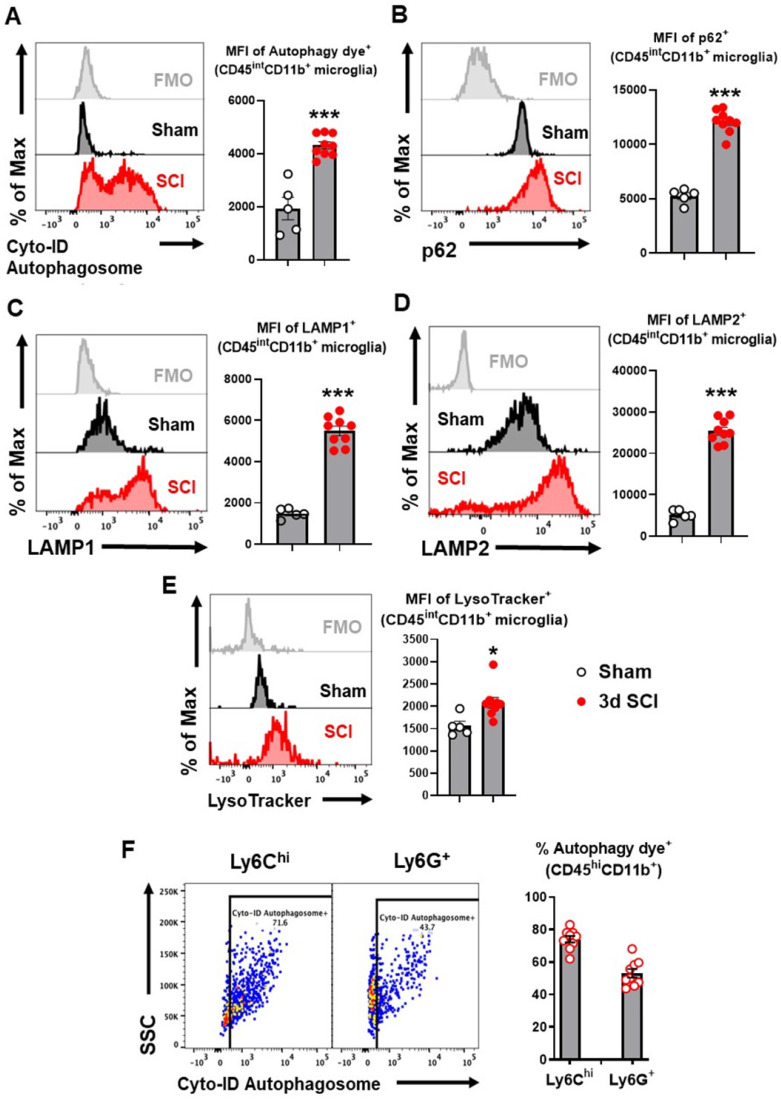
** Autophagy flux is inhibited in microglia and infiltrating monocytes in acute phase SCI.** Young adult male C57BL/6 mice were subjected to moderate contusion injury at T10 and flow cytometry was used to examine autophagy biomarkers at 3 days post-injury. (**A**) Representative histograms and mean fluorescent intensity (MFI) quantification show the relative production of autophagosomes in CD45^int^CD11b^+^ microglia as measured by the Cyto-ID Autophagy Detection Kit. (**B-D**) Representative histograms and MFI of p62/SQSTM1 (**B**), LAMP1 (**C**), and LAMP2 (**D**) in CD45^int^CD11b^+^ microglia. (**E**) Representative histograms and MFI of lysosomal activity in microglia as measured by the LysoTracker dye. (**F**) Representative dot plots and quantitative data depict the composition of infiltrating CD45^hi^CD11b^+^ monocytes with Cyto-ID positive staining in the spinal cord injury site. n = 5 mice for Sham group and 9 mice for 3 d SCI group. ***p < 0.001. Two-tailed unpaired t-test.

To explore autophagic flux in microglia/macrophages after SCI, we used flow cytometry to examine autophagy biomarkers in acute phase SCI. Representative plots demonstrating our gating strategy for the identification of CD45^int^CD11b^+^Ly6C^-^ CNS-resident microglia are shown in supplementary materials (**Figure S1**). At 3 days (3 d) after SCI, resident microglia within the lesion area showed significantly higher mean fluorescent intensity (MFI) when stained with the Cyto-ID Autophagy Detection Kit, a live cell dye for autophagic vacuoles (*p*<0.001, n=5 for sham, n=9 for SCI, **Figure 1A**). MFI of adapter protein p62 (SQSTM1), which mediates delivery of ubiquinated cargo to autophagosomes, was considerably increased within resident microglia at 3 d after injury compared with sham tissue (*p*<0.001, **Figure 1B**). As p62 is normally degraded with its cargo, its accumulation indicated inhibition of autophagy flux in microglia. Flow cytometry analysis for LAMP1 and LAMP2 also demonstrated significantly higher MFI within microglia (*p*<0.001, **Figure 1C-D**), signifying expansion of the lysosomal compartment after injury. LysoTracker, a fluorescent dye that is commonly used to qualitatively measure acidic organelles such as lysosomes, also displayed significantly higher levels after SCI (p<0.05, **Figure 1E**). Similarly, majority of Ly6C^high^ and Ly6G^+^ CD45^hi^CD11b^+^ myeloid cells infiltrating the injured spinal cord tissue were positive for autophagy dye (*p*<0.001, **Figure 1F**), indicating dysregulated autophagy in these cells.

**Figure 2:**
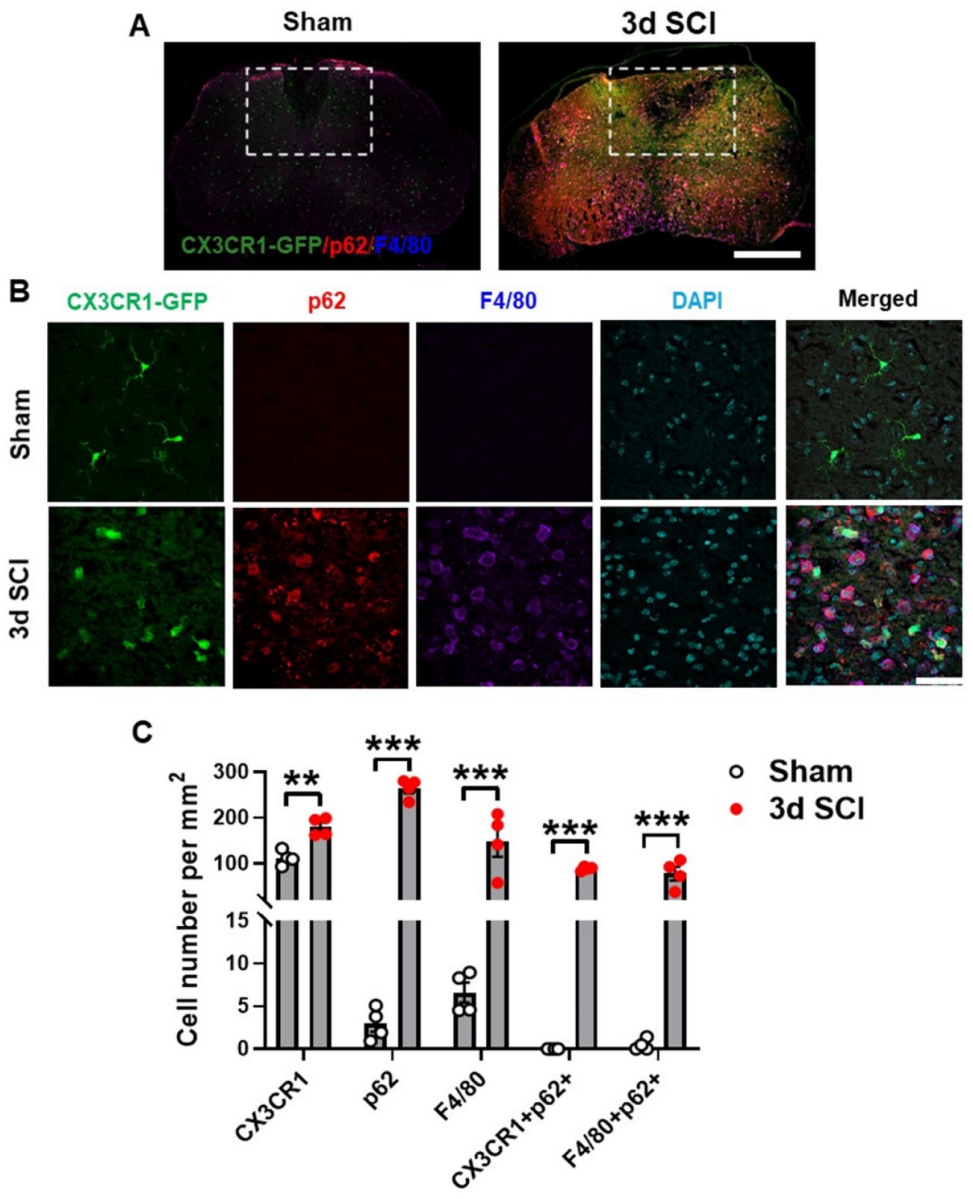
**Autophagosomes acutely accumulate in activated microglia and infiltrating macrophages at 3 days after SCI.** Young adult male *CX3CR1-GFP* mice underwent moderate contusion injury at T10. (**A**) Immunohistochemistry (IHC) representative images of GFP^+^(green)/p62^+^(red)/F4/80^+^ (blue) cells at 0.3 mm rostral to the epicenter. Insets display the dorsal white matter for quantification. (**B-C**) Representative images and cell count quantification of p62 (red) and F4/80 (blue) positive cells in the dorsal white matter of CX3CR1-GFP mice. n = 4 mice/group. *p < 0.05, **p < 0.01, ***p < 0.001. Two-tailed unpaired t-test. Scale bar = 500 µm (A) and 50 µm (B).

Presence of p62/SQSTM1 in microglia/macrophages was confirmed by immunohistochemistry (IHC) staining of sham (n=4) and 3 d injured (n=4) (**Figure 2A**) spinal cord sections from transgenic *CX3CR1-GFP* reporter mice 47 that expresses the fluorescent protein of enhanced GFP in microglia, monocytes, dendritic cells, and natural killer cells. Our studies demonstrated that SCI significantly increased the numbers of p62/SQSTM1-immunoreactive cells, which were highly co-localized with CX3CR1-GFP and with F4/80, a marker of infiltrating monocytes (**Figure 2B-C**). Additionally, we observed accumulation of the autophagosome marker microtubule-associated protein 1A/1B light chain 3 (LC3) (**Figure S2A-B**) in both CX3CR1-GFP and F4/80 positive cells, together confirming that autophagic flux is inhibited in microglia and infiltrating macrophages after SCI.

### Inhibition of autophagy exacerbates neuroinflammation after SCI

**Figure 3:**
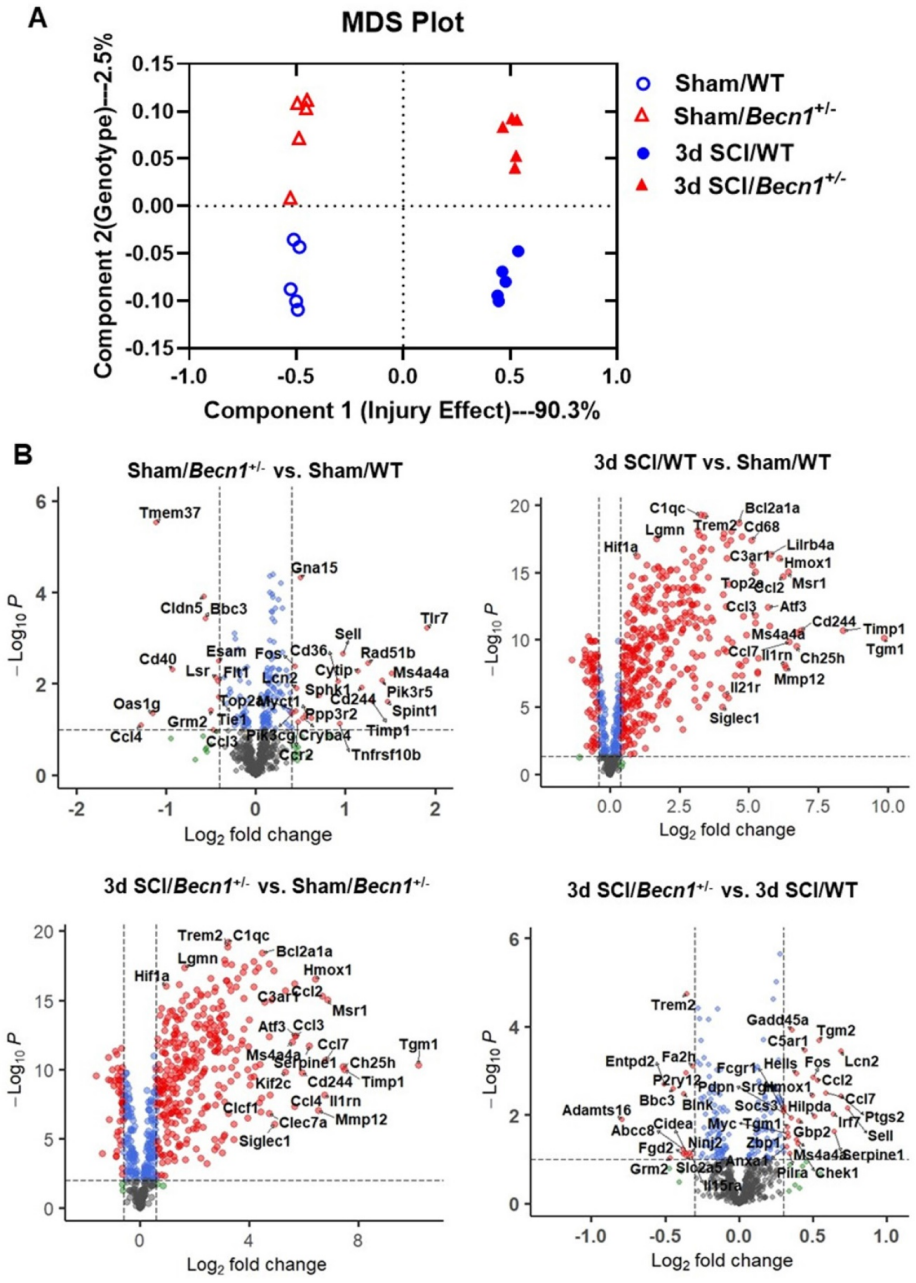
**Autophagy deficiency alters neuroinflammation transcriptome within the spinal cord at 3 days after SCI**. (**A**) Multi-dimensional scaling (MDS) was performed using all normalized gene counts from the NanoString neuroinflammation panel. (**B**) Volcano plot of genes in each set of pairwise comparisons with Log2 (fold change) and Log10(P). n = 5 mice/group.

To assess the effects of inhibited autophagic flux on neuroinflammation after SCI, we employed *Becn1*^+/-^ transgenic mice, a strain that is deficient in autophagy. To determine whether genotypic effects of *Becn1^+/-^* after SCI were associated with transcriptional changes, we evaluated gene expression in the spinal cord tissue of sham and 3 d SCI WT and *Becn1^+/-^* groups using NanoString's nCounter® technology (neuroinflammation panel). More than 700 genes were analyzed across three main themes of cellular function: Immunity and Inflammation (6 pathways); Neurobiology and Neuropathology (13 pathways); and Metabolism and Stress (4 pathways). Multiple dimension scale (MDS) of all normalized gene counts revealed a distinct separation of samples into individual groups across the first two principal dimensions (**Figure 3A**). The first dimension (x-axis) accounted for most of the variation (90.3%) across samples and separated the groups into two clusters by injury effect. The second dimension (y-axis) accounted for 2.5% of the transcriptome differences amongst all the genes examined and separated the groups by genotype. Interaction between sets of genes differentially expressed in each pairwise comparison was examined and presented as a Venn diagram (**Figure S3A**). SCI resulted in the upregulation of more than 400 genes examined by the neuroinflammation panel in both genotype groups. Autophagy deficiency in *Becn1^+/-^* groups resulted in altered expression of a group of genes in both Sham and SCI groups when comparing between genotypes (**Figure S3B**). In volcano plots (**Figure 3B**), pairwise comparison of the four different groups showed a large number of genes upregulated after SCI. In addition, we observed significant changes in inflammation and immune response related gene expression when comparing between the two genotype groups, with notable genes such as *Tmem37*, *Fos*, *Ccl7*, *Cd40*, *Tlr7*, and *Cd244*.

**Figure 4:**
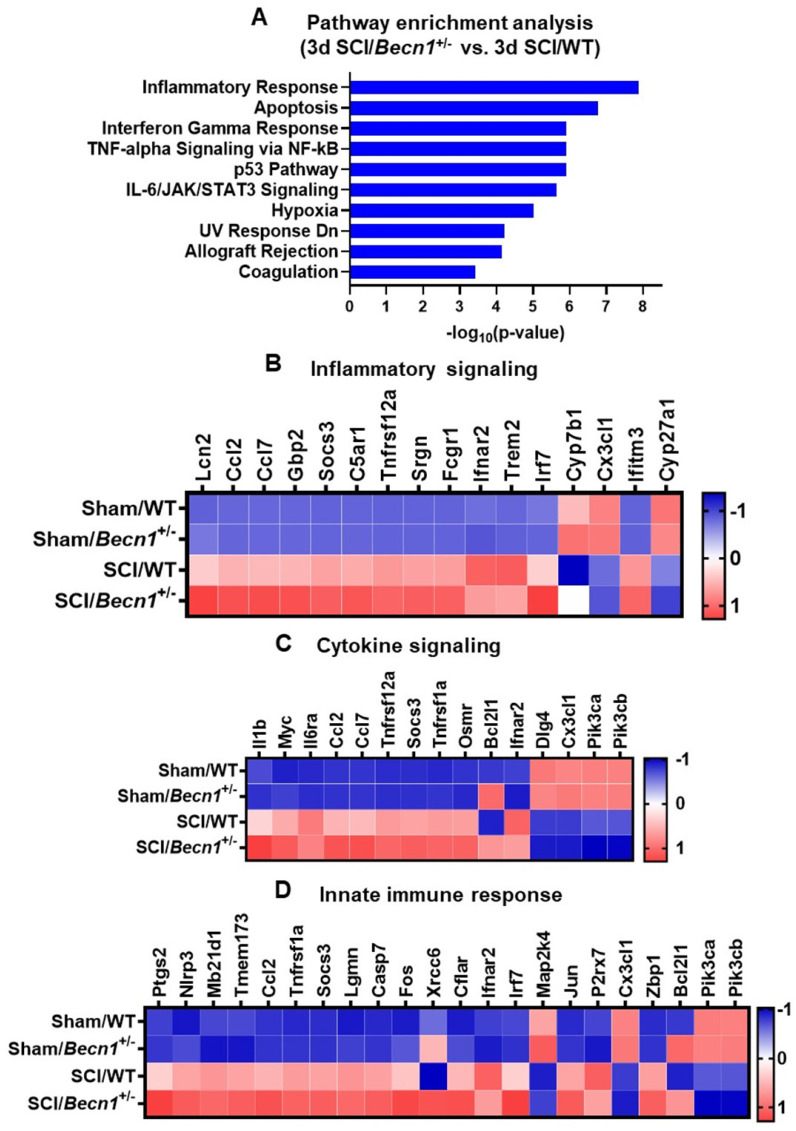
***Becn1^+/-^* mice display robust changes in signaling pathway and transcriptomes related to cellular function**. (**A**) Pathway enrichment analysis of genes modified by SCI in *Becn1^+/-^* vs. WT mice*.* (**B-D**) Depending on the percentage of genes within each pathway that has been modified, the top three pathways are Inflammatory response (**B**), Cytokine signaling (**C**), and Innate immune response (**D**). The DE genes contained in each pathway are normalized into z-scores and the group average was used for the heatmaps.

Pathway analysis of the differentially expressed genes in SCI/*Becn1*^+/-^ vs. SCI/WT using the Enrichr application showed inflammatory response as the most significantly enriched pathway in the Molecular Signatures Database (MSigDB) Hallmark 2020 (**Figure 4A**). Other enriched pathways included apoptosis, interferon gamma response, TNF-alpha signaling, p53 pathway, and IL-6/JAK//STAT3 signaling. Within the set of genes related to inflammatory signaling, expression levels of a total of 16 genes were modified by *Becn1^+/-^* in SCI groups, including upregulation of *Lcn2, Ccl2, Ccl7, and Gbp2* (**Figure 4B**). Fifteen genes related to cytokine signaling showed differential expression between SCI/*Becn1^+/-^* and SCI/WT, which included elevated *Il1β*, *Myc*, *Il6ra*, *Ccl2*, and *Ccl7* (**Figure 4C**). Further analysis identified genes related to innate immune response such as *Ptgs2*, *Nlrp3*, *Mb21d1*, *Tmem173*, *and Ccl2* as being upregulated by autophagy deficiency (**Figure 4D**). To further understand pathways affected by *Becn1^+/-^*, we also examined genes pertaining to autophagy, microglia and astrocyte function, and lipid metabolism, which yielded several genes of interest such as *Tgm2*, *Lamp1*, *Lamp2*, *Pink1*, *Apoe, Blnk, S100a10, Tgm1, Trem2, and Atg9a* (**Figure S4**).

**Figure 5:**
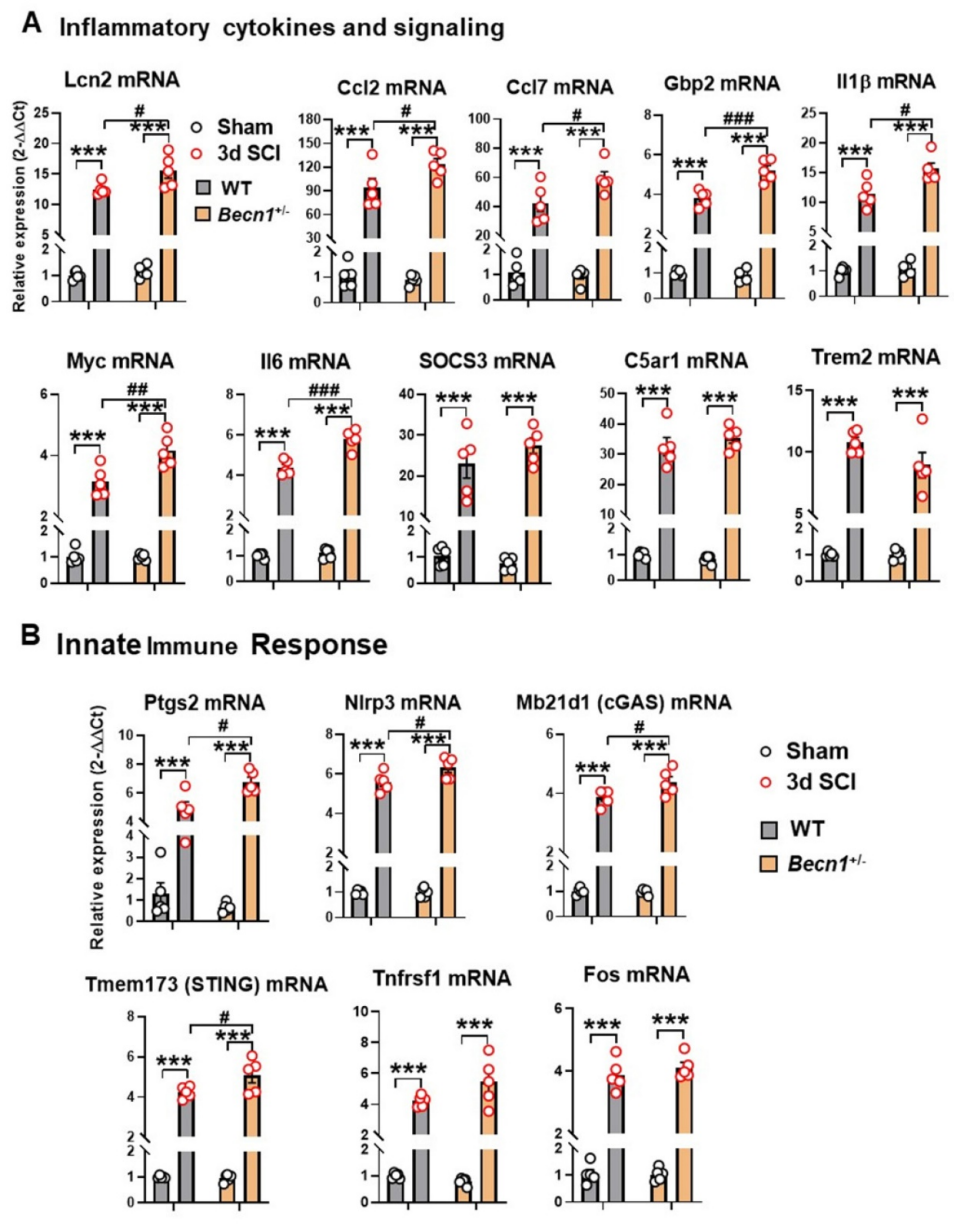
** The effects of autophagy deficiency on the inflammatory signaling and innate immune genes in the spinal cord at 3 d post-injury.** (**A**) Quantitative real-time PCR revealed that a total of seven pro-inflammatory markers showed significantly higher levels in *Becn1^+/-^* mice compared to WT littermates at 3d after SCI. (**B**) Several innate immune genes (Ptgs2, Nlrp3, Mb21d1, and Tmem173) showed significant injury effects between Sham and SCI groups but increased even further in the spinal cord of SCI/*Becn1*^+/-^ mice. n = 5 mice/group, ***p < 0.001 vs. Sham groups; ^#^*p* < 0.05,^ ##^*p* < 0.01, ^###^*p* < 0.001 vs. SCI/WT. Two-way ANOVA followed by Tukey's multiple comparison.

To further validate the findings of NanoString array and gain more insight into the inflammatory response after SCI, we used qPCR to examine both pro- and anti-inflammatory marker levels in the injury site tissue. Of these, 7 genes involved in cytokine signaling (*Lcn2*, *Ccl2*, *Ccl7*, *Gbp2*, *Il1β*, *Myc*, and *Il6*) showed significant upregulation after injury in both genotypes but increased even further in the spinal cord of SCI/*Becn1^+/-^* mice (**Figure 5A**). Although all 3 genes (*SOCS3*, *C5ar1*, and *Trem2*) showed marked increase after injury, no differences were observed between the genotypes. Interestingly, four innate immune response genes, *Ptgs2*, *Nlrp3*, *Mb21d1*, and *Tmem173*, showed significantly higher levels in SCI/*Becn1^+/-^* than SCI/WT, indicating a more exaggerated innate immune response in *Becn1^+/-^* mice after injury (**Figure 5B**). Both *Tnfrsf1* and *Fos* showed similar trends between WT and *Becn1^+/-^* groups at 3 d after SCI. Taken together, our results show potentiation of neuroinflammation in autophagy deficient mice after SCI.

### Autophagy deficiency potentiates pro-inflammatory signaling

**Figure 6:**
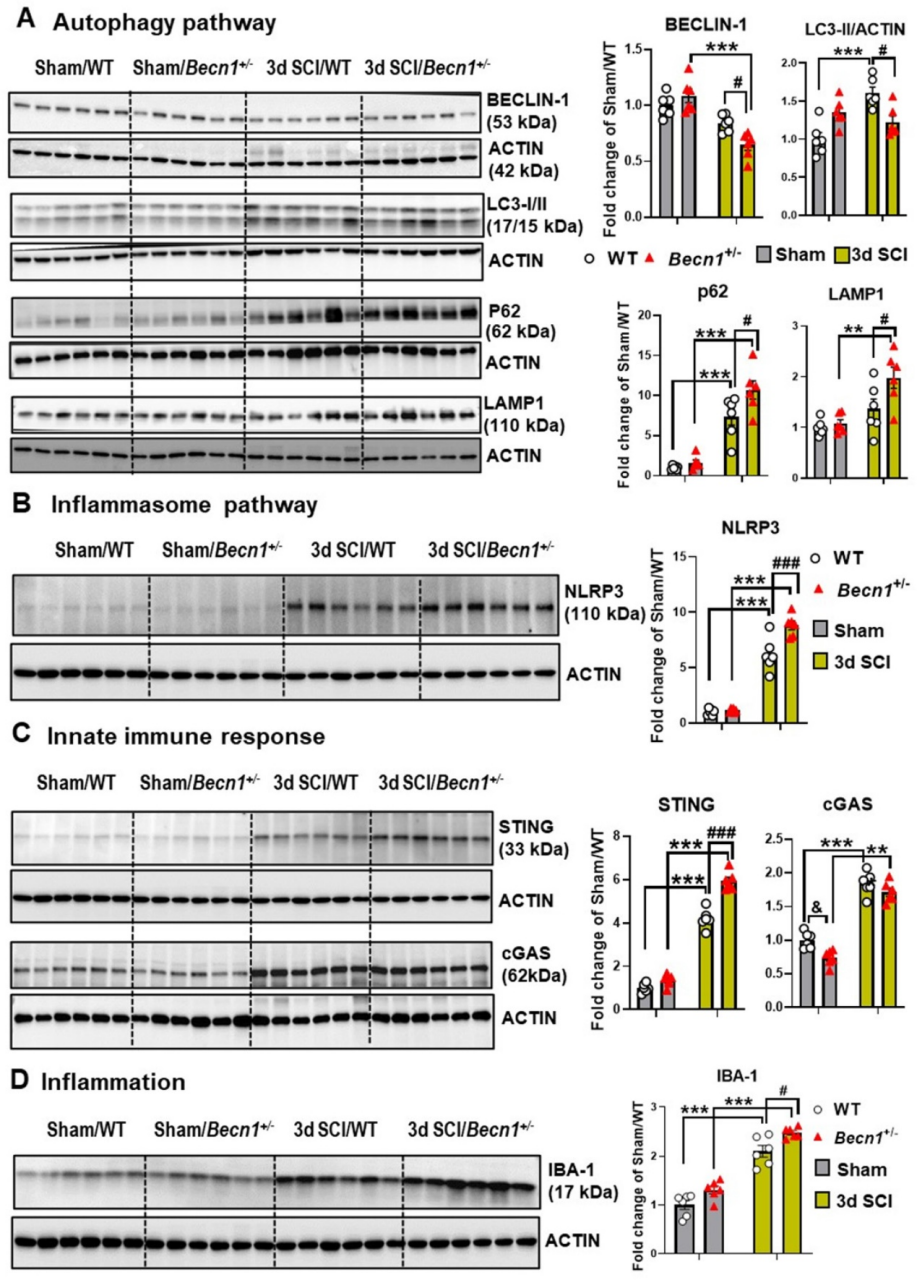
**Effects of autophagy deficiency on protein expression level of key inflammatory and autophagic markers.** Young adult male *Becn1^+/-^* and WT mice were subjected to moderate contusion injury and Western blotting was used to examine autophagic and inflammatory markers at 3 d post-injury. (**A**) Expression of BECLIN-1, p62, the autophagosome marker LC3-II and the lysosome marker LAMP1. (**B**) Expression of inflammasomes NLRP3. (**C**) Expression of the markers cGAS and STING for innate immune response following SCI. (**D**) Expression of microglia/macrophages marker IBA-1. n = 6 mice/group, *p < 0.05, ***p < 0.001 vs. Sham/WT. ^#^*p* < 0.05, ^##^*p* < 0.005, ^###^*p* < 0.001 vs. SCI/WT. Two-way ANOVA followed by Tukey's multiple comparison.

To further investigate the effects of autophagy deficiency on inflammatory signaling after SCI, we examined levels of proteins involved in the regulation and formation of autophagosomes as well as in neuroinflammatory pathways. There were no differences in all markers examined by protein level between sham groups (**Figure 6A**). At 3 d post-injury, Western blot analysis of BECLIN-1 showed significantly lower levels in *Becn1^+/-^* mice than those in WT group. In agreement with previous studies 3-5, SCI in WT mice caused increased protein levels of LC3-II, p62/SQSTM1, and LAMP1 (n=6 mice/group). Autophagy deficiency in *Becn1^+/-^* mice led to further accumulation of p62 and LAMP1 at 3 d post-injury when compared to SCI/WT animals, suggesting impaired lysosomal function in the spinal cord tissue surrounding the injury site. Reduced expression level of LC3-II observed in SCI/*Becn1^+/-^* mice supports the notion that Beclin-1 deficiency impairs the formation of autophagosomes 18. Examination of inflammation-related markers showed that autophagy deficiency led to significant elevation of the inflammasome marker NLRP3 (**Figure 6B**), innate immune response markers STING and cGAS (**Figure 6C**), and microglia/macrophages marker IBA-1 (**Figure 6D**), suggesting exacerbation of innate immune responses following SCI.

To assess the effects of sex, we examined the same autophagy markers and inflammatory pathways in both male and female mice (**Figure S5**). There were no differences in the protein expression level of any markers between two sham groups. Although no significant injury effects were observed in Beclin-1 expression for males at 3 d after injury, female mice showed a significant decrease. Elevated LC3-II expression was observed in SCI/male mice but not in females. Furthermore, p62 showed significantly lower expression levels in SCI/Female compared to SCI/Male. There were no differences in LAMP1 between sexes. We also examined the levels of the inflammasome marker NLRP3 and innate immune response markers cGAS and STING, but no significant sex differences were observed in these markers.

**Figure 7:**
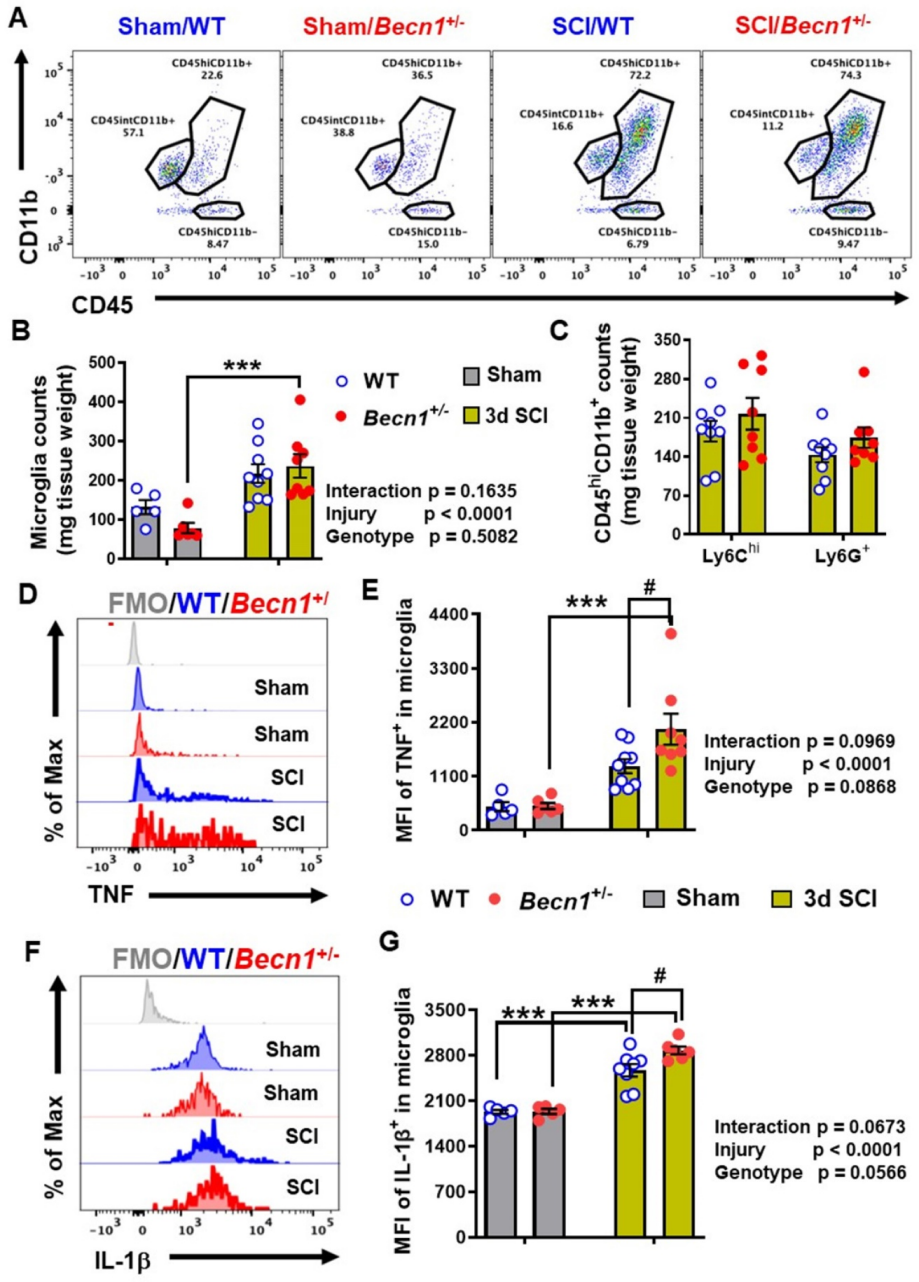
** The effects of autophagy deficiency in *Becn1^+/-^* mice on the neuro-immune response in the spinal cord at 3 d post-injury.** (**A-C**) Flow cytometry analysis showed increased numbers of CD45^int^CD11b^+^ microglia and CD45^hi^CD11b^+^ leukocyte infiltration in both WT and *Becn1^+/-^* mice following SCI. Representative dot plot of immune cells in the spinal cord of sham and injured mice are shown in A. Quantification of CD45^int^CD11b^+^ microglia counts and CD45^hi^CD11b^+^ leukocyte are indicated in B and C. (**D-G**) Proinflammatory cytokines TNF (D-E) and IL-1β (**F-G**) in the microglia showed significant increase after SCI, with even higher levels in *Becn1^+/-^* mice. n = 5 (Sham/WT), 9 (SCI/WT), 6 (Sham/*Becn1^+/-^*), and 8 (SCI/*Becn1^+/-^*) mice. ***p < 0.001 vs. Sham groups. ^#^*p* < 0.05 vs. SCI/WT. Two-way ANOVA followed by Tukey's multiple comparison.

At 3 days post-injury, flow cytometry was used to examine neuroinflammation in the impact site. Our data showed increased numbers of CD11b^+^CD45^int^ microglia and CD11b^+^CD45^hi^ leukocyte infiltration in both WT and *Becn1^+/-^* mice following SCI (n=5-9 mice/group). Microglial and leukocyte cell numbers were not altered between SCI/*Becn1^+/-^* and SCI/WT mice when normalized by tissue weight (**Figure 7A-C**). However, our analysis showed marked increase in the expression levels of the pro-inflammatory cytokine TNF in microglia derived from *Becn1^+/-^* mice as compared to WT animals after injury (**Figure 7D-E**). Furthermore, IL-1β showed marked increase in both genotype groups after injury when compared to sham surgery groups and was further upregulated in microglia form SCI/*Becn1^+/-^* as compared to SCI/WT littermates (**Figure 7F-G**), indicating exacerbated pro-inflammatory phenotype. In addition, SCI resulted in increased MFI of TNF and IL-1β in Ly6C^hi^ monocytes and Ly6G^+^ neutrophils infiltrating the spinal cord in both WT and *Becn1^+/-^* mice. However, our data showed no differential change between the genotypes at 3 d post-injury (**Figure S6**), suggesting that there is no genotype-based difference in infiltrating myeloid compartment. Together these findings show that autophagy deficiency potentiates pro-inflammatory activation of the cytokines in microglia leading to exacerbation of inflammation.

### *Becn1*^+/-^ mice exhibits worse functional recovery and tissue damage after SCI

**Figure 8:**
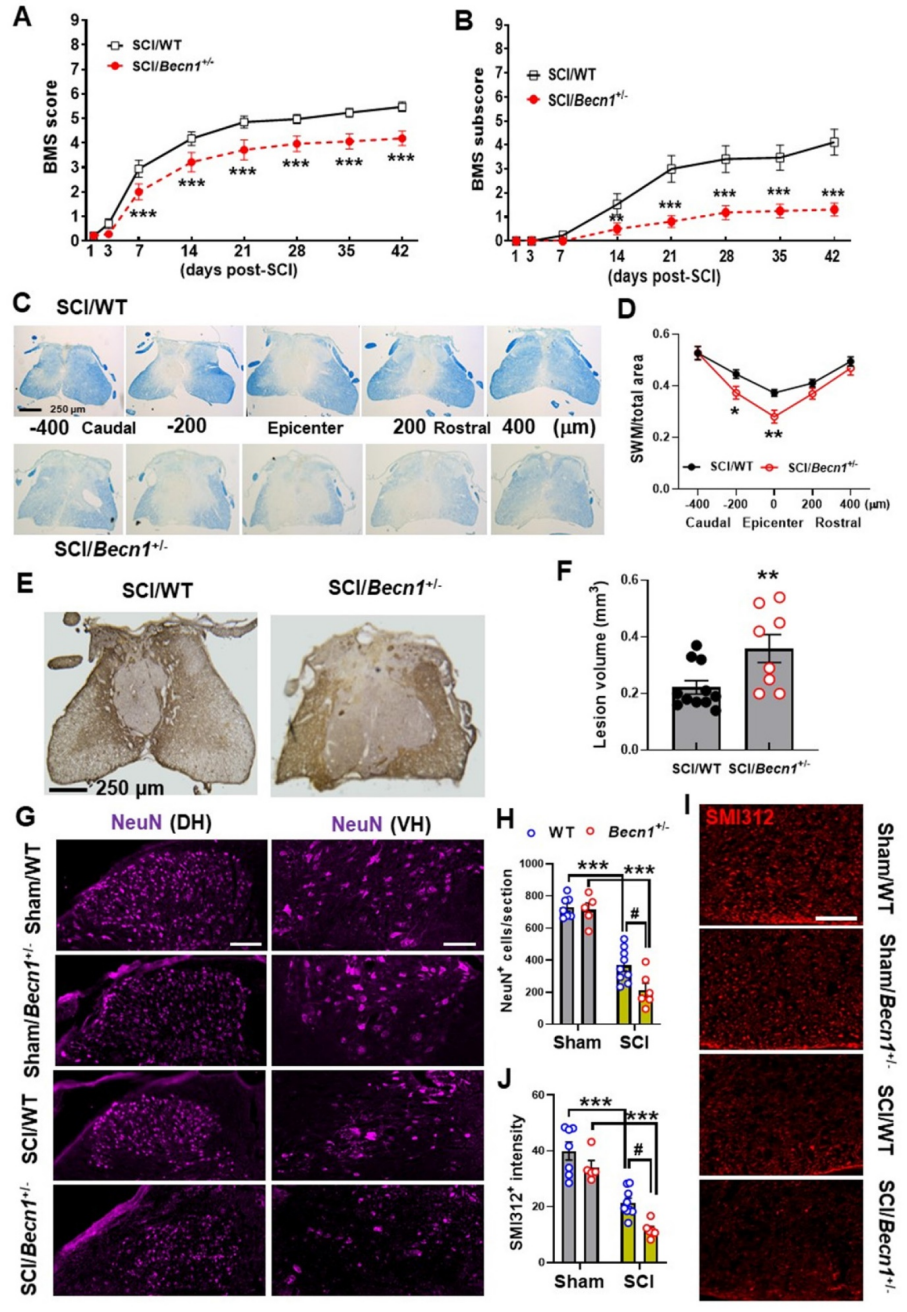
**Effects of autophagy deficiency on long-term functional outcome and tissue loss**. (**A-B**) BMS and subscores for WT and *Becn1^+/-^* mice at weekly assessment of hindlimb motor function. Two-way repeated measurement ANOVA followed by Holm-Sidak's post-hoc test. n=17 (SCI/WT) and 16 mice (SCI/*Becn1*^+/-^). **p < 0.01, ***p < 0.001 vs. SCI/WT. (**C-D**) Representative images and quantification of spared white matter (SWM) at 6 w post-injury. n=11 (SCI/WT) and 8 mice (SCI/*Becn1^+/-^*). *p < 0.05, **p < 0.01 vs. SCI/WT. Two-way ANOVA followed by Tukey's multiple comparison. (**E-F**) Representative images of GFAP-DAB staining and quantification of the lesion volume at 6 w SCI. n=11 (SCI/WT) and 8 mice (SCI/*Becn1^+/-^*). **p < 0.01 vs. SCI/WT. Two-tailed unpaired t-test. (**G-H**) Representative images of NeuN (purple) staining at the ventral (VH) and dorsal horn (DH) and quantification in the grey matter **(I-J)** Representative images and quantification of the neurofilament marker SMI312 (red) in the white matter, Scale bars = 100 μm (**G**) and 50 μm (**I**). n = 7 (Sham/WT), 5 (Sham/*Becn1*^+/-^), 9 (SCI/WT), and 6 (SCI/*Becn1*^+/-^) mice/group. ***p < 0.001 vs. Sham groups; ^#^*p* < 0.05 vs. SCI/WT. Two-way ANOVA followed by Tukey's multiple comparison.

To fully examine functional outcome of SCI under conditions of autophagy deficiency, we used BMS to assess hindlimb locomotor functions. Records from weekly assessment of BMS scores and subscores showed that SCI/*Becn1*^+/-^ mice were recovering at a slower rate than their WT littermates (**Figure 8A-B**). Starting from as early as 7 days post-injury, the average score for the SCI/WT mice was 2.941±0.189, indicating that most WT mice within the group showed extensive ankle movement and plantar placement of the hind paw. At the same time point SCI/*Becn1^+/-^* mice had an average score of 2.000±0.195, which is indicative of only ankle movement but no plantar placement. This dramatic genotype difference at 1 week post-injury (n=17 for WT, n=16 for *Becn1^+/-^*, *p*= 0.003) was the start of a persistent trend that would continue for six weeks (*p*<0.001) at which point motor function recovery appeared to have reached a plateau point for both injury groups. We detected a significant main effect of genotypes [F_(1, 31)_=8.079, *p*=0.0079 for BMS scores and F_(1, 31)_=14.03, *p*=0.0007 for BMS subscores]. After 6 w of injury, we examined tissue damage after injury by spared white matter (SWM) and lesion volume (LV). Luxol Fast Blue staining showed significantly lower area of SWM in *Becn*1^+/-^ mice at the epicenter compared to their WT counterparts (**Figure 8C-D**). These results are congruent with GFAP staining and quantification of lesion volume by unbiased stereology showed a much larger area of glial scarring in *Becn1*^+/-^ compared to their WT littermates (**Figure 8E-F**). Assessment of survival neurons in the grey matter of the injured spinal cord showed aggravated loss in IHC staining of NeuN+ cells in *Becn1* deficient mice (**Figure 8G-H**). We also observed an amplified decrease of neurofilaments in the surrounding white matter of SCI/*Becn1*^+/-^ mice (**Figure 8I-J**). To preclude the possibility of baseline genotypic effects on motor and cognitive function (**Supplementary Material**), we examined spontaneous activity via open field (OP) test, through which we found no difference between *Becn1*^+/-^ and their WT littermates (**Figure S7A**). Assessment of cognitive function using Y-maze and novel object recognition tests demonstrated no differential changes of the performance between two genotypes (**Figure S7B**). Together these findings demonstrate that genetic autophagy deficiency exacerbates SCI-mediated motor function deficit which is associated with potentiated tissue damage.

### Enhanced autophagy attenuates SCI injury

**Figure 9:**
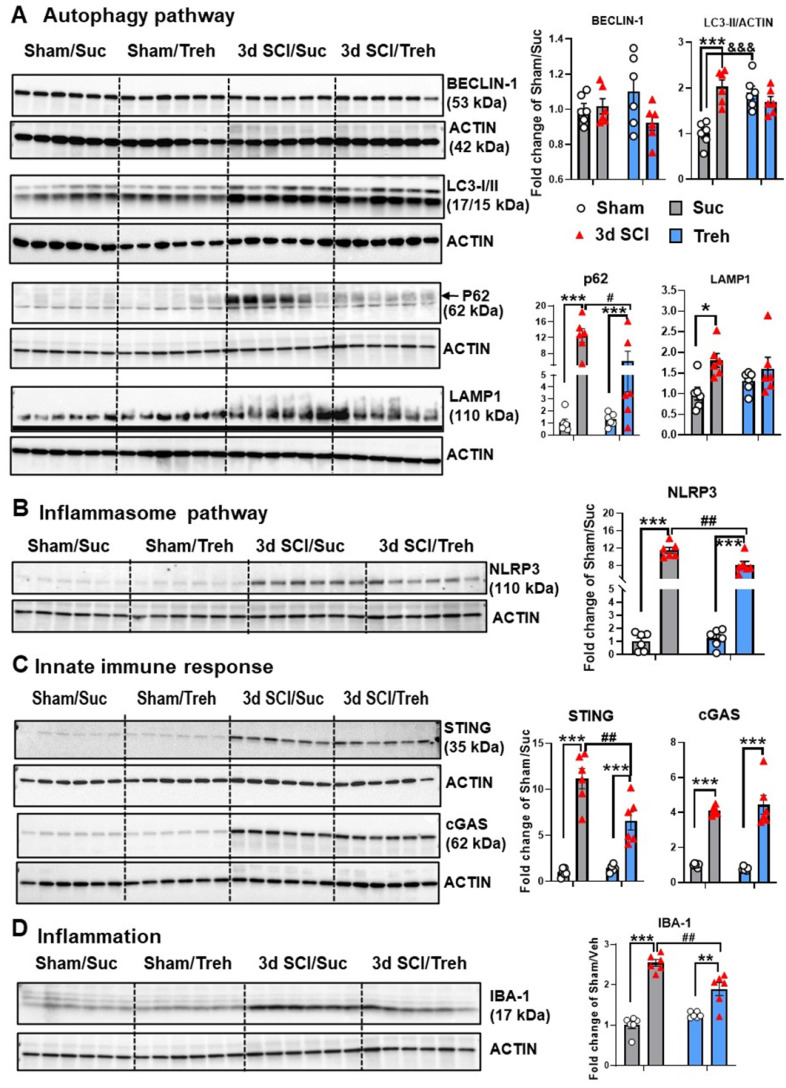
**Effects of Trehalose treatment on protein expression level of key inflammatory and autophagic markers.** Young adult C57BL/6 mice subjected to moderate contusion injury were given 5% trehalose or sucrose via oral gavage twice per day in addition to treated drinking water. At 3 d after SCI, spinal cord tissue surrounding injury site were dissected for examination of the autophagic and inflammatory markers. Western blot analysis of protein expression for BECLIN-1, LC3-II, p62, and LAMP1 (**A**), NLRP3 (**B**), cGAS and STING (**C**), and IBA-1 (**D**) and the representative blot images are shown. n = 6 mice/group. * *p* < 0.05, **p < 0.05, ***p < 0.001 vs. Sham groups; ^#^*p* < 0.05, ^##^*p* < 0.01 vs. SCI/WT; ^&&&^ *p* <0.001 vs. Sham/Suc group. Two-way ANOVA followed by Tukey's multiple comparison.

Given the robust effects of genetic autophagy deficiency on inflammatory markers, we investigated whether treatment with an autophagy enhancer could attenuate acute phase inflammation and long-term functional outcomes. For acute studies, mice were given 5% trehalose or sucrose (control) via oral gavage twice per day in addition to treated drinking water. At 3 d SCI, Western blot analysis did not show significant changes in the expression level of BECLIN-1 (n=6 mice/group, **Figure 9A**). However, we found significant decrease of p62 levels in SCI/Trehalose (SCI/Treh) mice compared to SCI/Sucrose (SCI/Sucr) animals (n=6 mice/group, *p*=0.0352), indicative of improved autophagy flux after trehalose treatment. Other autophagy markers, LC3-II, appeared significant increase in Sham/Treh animals (n=6 mice/group, *p*=0.0004) compared to Sham/Suc group. However, the marked increase of LC3-II at 3 d after injury that we see between Sham/Suc and SCI/Suc groups (*p*<0.0001) was not present in trehalose treatment groups. There were no differences in LAMP1 expression between SCI groups. For a better picture at the state of autophagy flux, the significant decrease of p62 levels in SCI/Treh mice compared to SCI/Suc animals (*p*=0.0352) is indicative of improvement after trehalose treatment. The inflammasome related protein NLRP3 was significantly decreased in SCI/Treh compared to SCI/Suc (*p*=0.0025, **Figure 9B**). In addition, trehalose treatment significantly reduced protein levels of innate immune response marker STING (*p*=0.0016) and microglia/macrophages marker IBA-1 (*p*=0.001, **Figure 9C-D**). These results suggest that trehalose could restore autophagic flux and reduce inflammatory responses following SCI.

**Figure 10:**
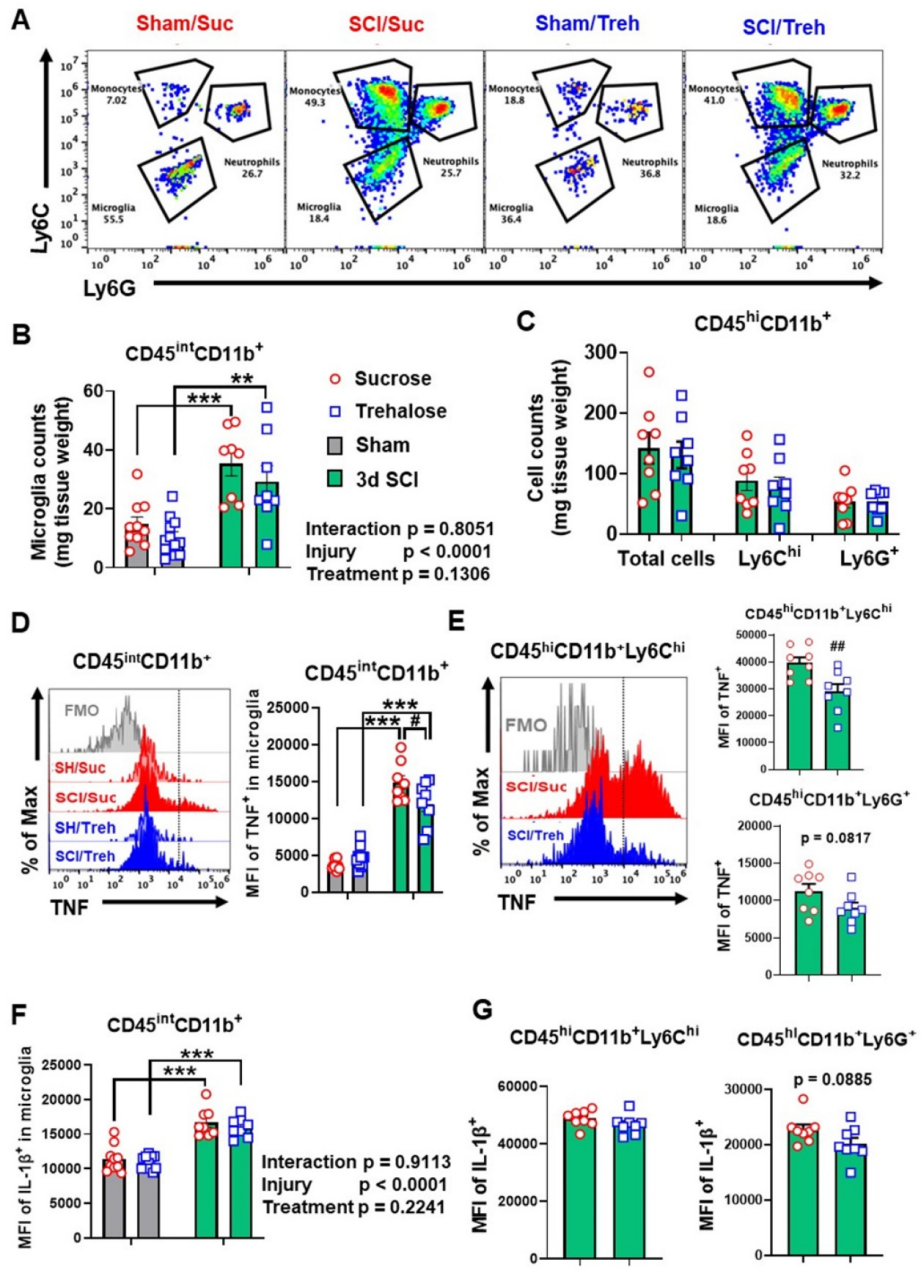
** The effects of Trehalose treatment on the neuro-immune response of the spinal cord at 3 d post-injury.** (**A-C**) Flow cytometry analysis showed similar numbers of CD45^int^CD11b^+^ microglia and CD45^hi^CD11b^+^ leukocyte infiltration in both sucrose (Suc) and trehalose (Treh) groups following SCI. Representative dot plot of immune cells in the spinal cord of sham and injured mice are shown in A. Quantification of CD45^int^CD11b^+^ microglia counts and CD45^hi^CD11b^+^ leukocyte are indicated in B and C. (**D-G**) Proinflammatory cytokine TNF (D-E) in microglia and monocytes showed significant decrease in Trehalose group after SCI, while IL-1β (F-G) remained the same between Trehalose and Sucrose. n = 10 (Sham/Suc), 8 (SCI/Suc), 12 (Sham/Treh), and 8 (SCI/Treh) mice. ** *p* < 0.01, *** *p* < 0.001 vs. Sham groups. # *p* < 0.05, ## p < 0.01 vs. SCI/Suc. Two-way ANOVA followed by Tukey's multiple comparison.

Further analysis of acute stage trehalose treatment through the use of flow cytometry showed a significant increase of CD45^int^CD11b^+^ microglia in both sucrose and trehalose treated mice at 3 d SCI (n=8-10/group). No differences were observed in microglia and infiltrating leukocyte cell numbers between SCI/Treh and SCI/Suc animals when we normalized the results by tissue weight (**Figure 10A-C**). However, the MFI of TNF expressed in microglia (*p*=0.0309) and CD45^hi^CD11b^+^Ly6C^hi^ monocytes (*p*=0.0078) appeared significant decrease in SCI/Treh mice compared to SCI/Suc (**Figure 10D-E**). Finally, no differences between treatment groups were observed for the cytokine IL-1β (**Figure 10F-G**).

**Figure 11:**
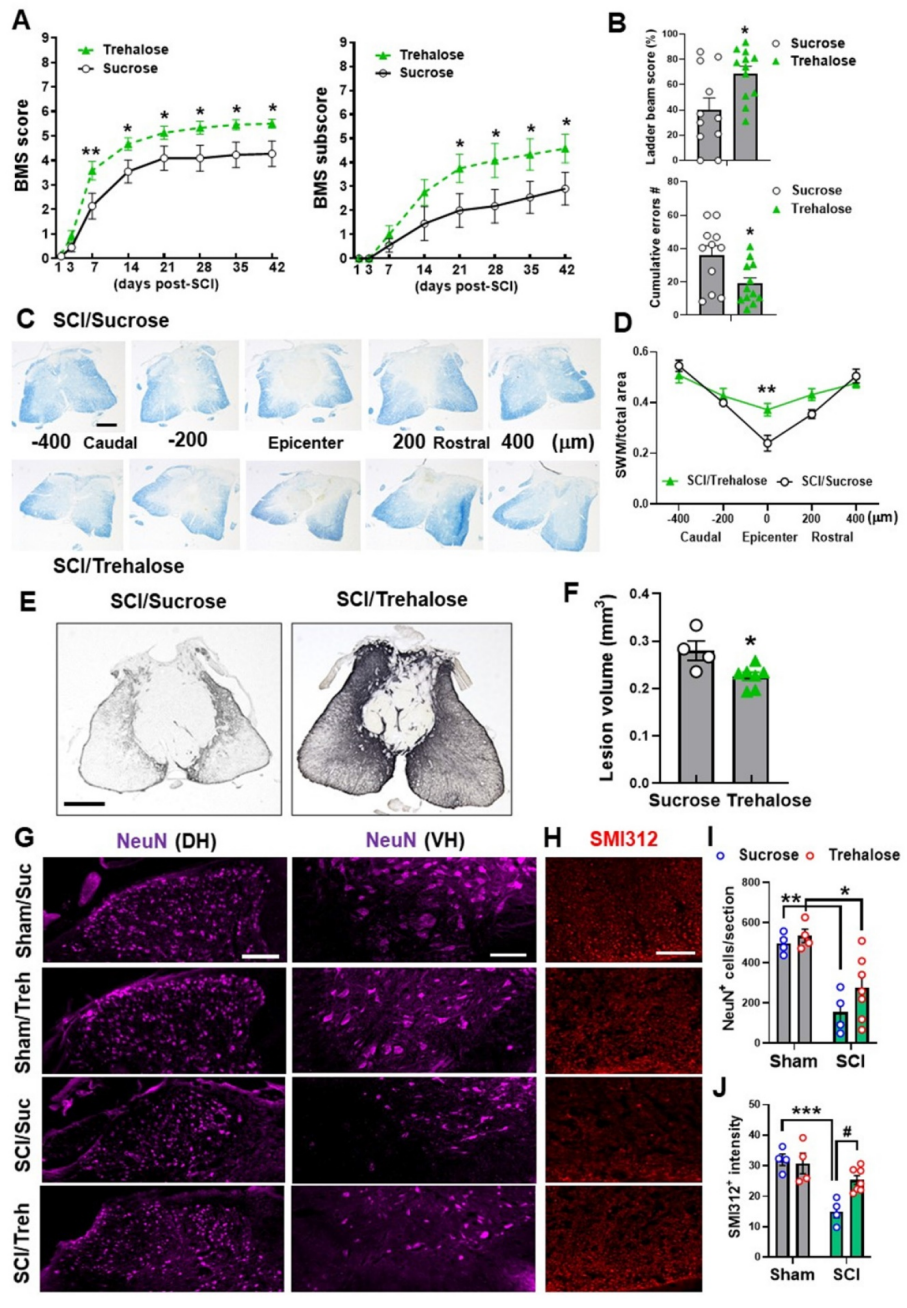
** Effects of Trehalose on long-term functional outcomes following SCI.** Young adult C57BL/6 mice were administrated 5% trehalose or sucrose via oral gavage twice per day for the first week followed by continuous administration at 2.5% trehalose or sucrose in their drinking water for 6 w. (**A-B**) BMS and subscores (A) showed significantly better recovery of their hindlimb motor function in trehalose-treated mice compared to sucrose-treated animals. Trehalose treated group showed significantly higher ladder beam scores and lower number of cumulative errors compared to sucrose control group in horizontal ladder test (B). n = 11 (sucrose) and 12 mice (trehalose). * *p* < 0.05, ** *p* < 0.01 vs. SCI/Sucrose group. (**C-D**) Representative images and quantification of SWM at 6 w post-injury. n = 4 (SCI/Sucrose) and 7 mice (SCI/Trehalose). ** *p* < 0.01 vs. SCI/Sucrose group. Scale bars = 250 μm. (**E-F**) Representative images of GFAP-DAB staining and quantification of the lesion volume at 6 w SCI. n = 4 (SCI/Sucrose) and 7 mice (SCI/Trehalose). * *p* < 0.05 vs. SCI/Sucrose. Scale bars = 250 μm. (**G-J**) Immunohistochemistry (IHC) representative images of NeuN (G, purple) in the dorsal (DH) and ventral horn (VH) regions of the spinal cord at 6w after injury. H indicates representative images of the neurofilament marker SMI312 (red) in the surrounding white matter. Quantification of NeuN+ cells in the grey matter **(I)** and the SMI312+ intensity in the white matter (J) are presented. Scale bars = 100 μm (**G**) and 50 μm (**H**). n = 4 (Sham/Suc), 4 (Sham/Treh), 4 (SCI/Suc), and 7 (SCI/Treh) mice/group. * *p* < 0.05, ** *p* < 0.01, *** *p* < 0.001 vs. Sham groups; ^#^ *p* < 0.05 vs. SCI/Suc group. Two-way repeated measurement ANOVA followed by Holm-Sidak's post-hoc test for A, Mann Whitney test for B (upper panel) and D, unpaired t test for B (low panel) and F, Two-way ANOVA followed by Tukey's multiple comparison for I-J.

For chronic studies, mice were administrated 5% trehalose or sucrose via oral gavage twice per day for the first week followed by continuous administration at 2.5% trehalose or sucrose in their drinking water for 6 w. Weekly assessment of BMS and BMS subscores demonstrated that trehalose-treated mice showed significantly better outcomes as compared to control-treated animals** (Figure 11A)**. We detected a significant main effect of drugs [F_(1, 168)_=31.04, *p*<0.0001 for BMS scores and F_(1, 168)_=17.41, *p*<0.0001 for BMS subscores]. To assess the effects of trehalose on motor coordination and skilled walking, horizontal ladder walk was performed at 6 w post-injury. Ladder beam score and the number of stepping errors showed significantly improved outcomes in trehalose-treated mice compared to sucrose-treated animals (**Figure 11B**). Histological assessment of the spinal cord at 6 w showed significantly higher area of SWM in SCI/Treh mice at the epicenter compared to the sucrose treated control group (**Figure 11C-D**). Subsequent staining with GFAP and quantification of the lesion volume also showed a much smaller area of glial scarring after prolonged treatment with trehalose (**Figure 11E-F**). To assess the effects of trehalose treatment on pathological loss of neurons and damage to neurofilaments, we used IHC to stain NeuN (**Figure 11G**) and SMI312 (**Figure 11H**). In the grey matter, significant neuronal loss was observed in both groups after SCI, with no significant difference between treatment methods (**Figure 11I**). However, quantification of neurofilament staining in the white matter showed significantly higher intensity for SCI/Treh compared to SCI/Suc groups (**Figure 11J**). Together these results indicate that pharmacological enhancement of autophagy mitigates neuroinflammation, tissue damage, and motor function deficits following SCI.

## Discussion

In the present study, we investigated the role of autophagy in neuroinflammation after SCI. We demonstrated that autophagic flux was inhibited in microglia and infiltrating myeloid cells during the acute stage of traumatic SCI. This was reflected by increased LC3-positive microglia/macrophages within the spinal cord indicating accumulation of autophagosomes in these cells. The increased accumulation of p62/SQSTM1 also demonstrated that degradation of autophagosomes by lysosome has been inhibited, causing the autophagy flux to be dysfunctional. Furthermore, the autophagy hypomorph transgenic *Becn1^+/-^* mice were used to examine transcriptional and functional responses following SCI. Transcriptome changes assess by NanoString neuroinflammation panel reflected increase in pro-inflammatory gene expression in *Becn1^+/-^* as compared to WT mice in response to SCI. Potentiated neuroinflammation on these autophagy-deficient mice, was associated with exacerbated long-term functional deficits and tissue damage. Moreover, continuous treatment with autophagy enhancer trehalose was able to significantly re-establish autophagic flux, decrease neuroinflammatory markers and tissue damage, and improve motor function recovery after SCI. Taken together, genetically inhibiting autophagy induced pro-inflammatory responses to injury, with detrimental effects on tissue damage and motor functional recovery after SCI; pharmacologically enhancing autophagy attenuated pro-inflammatory phenotypes, with beneficial effect on functional recovery.

We and others previously reported 1, 2 that autophagy flux is inhibited in spinal cord tissue after SCI in both rat and mouse models and inhibition of autophagy after SCI occurs in several cell types including CD11b^+^ microglia and infiltrating cells 3. Thus, activation of microglia/monocytes after SCI is associated with inhibition of autophagy flux. The current study has now captured similar changes in microglia/macrophages during the acute phase of SCI using a combination of ex *vivo* cellular assays and histological approaches. At the cellular level, we observed accumulation of an autophagosome-specific dye and p62 in both microglia and infiltrating monocytes after SCI. The present study is among the first to examine these changes at the cellular level in SCI model. Immunofluorescence analysis using F4/80 antigen expressed primarily on macrophages combined with CX3CR1-GFP microglial reporter mice confirmed immuno-reactivity of LC3 and p62 in both CX3CR1^+^ and F4/80^+^ cells in injured spinal cord. While elevated LC3 signal in both activated microglia and infiltrating macrophages shows more autophagosomes, the overlap between LC3 and p62 accumulation in both cells is more prominent, strongly indicating decrease in autophagic degradation and impairment of autophagy flux.

To determine the causal effect and relationship between autophagy and inflammatory responses to SCI, we used the autophagy hypomorph *Becn1^+/-^* mice. Using the NanoString Neuroinflammation Panel, we demonstrated that SCI caused transcriptional activation of more than 400 neuroinflammation-related genes in the spinal cord, and 20 genes are *Becn1^+/-^*-related. Transcriptome changes show increased expression of genes related to inflammation signaling, such as *Lcn2, Ccl2, Ccl7, Gbp2, Il1β, Il6ra, and Myc* in SCI/*Becn1^+/-^* mice as compared to SCI/WT animals. Lcn2 (Lipocalin 2) is an iron-trafficking protein involved in multiple processes such as apoptosis and innate immunity 48. Several studies point to Lcn2 as a chemokine inducer that plays an important role in neuropathic pain and is detrimental to SCI recovery 49, 50.

The genes *Ccl2* and *Ccl7* both encode chemokines that have been reported to be involved in cell migration of leukocytes to the injury sites in the CNS in several disease models 51. Recent SCI studies demonstrated a role for CCL2 in activating microglia and recruitment of T cells in the injured spinal cord 52, 53. Further studies also implicated that both chemokines play a significant role in regulating neuropathic pain and opioid-induced analgesia after sciatic nerve injury and SCI 54, 55. Another gene in the inflammatory signaling pathway, *Gbp2,* encodes the Interferon-Induced Guanylate-Binding Protein 2, which is part of Guanylate binding protein family and has significant roles in pattern recognition, interferon induced inflammatory signaling and pyroptosis 56-58. Although studies for this protein are mostly limited to immunology, it could also be a potential target for studying neuroinflammation after SCI. The gene *Il1β* encodes a well-known pro-inflammatory cytokine that regulates glial activation and recovery after SCI 59-61. The gene *Il6ra* encodes IL-6 receptor alpha, which regulates downstream signaling for the cytokine IL-6 in SCI models 62. Finally, the gene *Myc* has been found to be involved with neuroprotective signaling mechanisms and brain-derived neurotrophic factor pathway 63. Innate immune response genes *Ptgs2* (COX2), *Nlrp3*, *Mb21d1* (cGAS), and *Tmem173* (STING) include several genes known to be involved in SCI. The gene *Ptgs2* encodes Prostaglandin-endoperoxide synthase 2, which is also known as COX2. Studies show that COX2 expression is upregulated within endothelial cells after SCI 64 and plays a significant role in mediating inflammation for astrocytes 65. The gene *Nlrp3* is a key player in inflammasome formation, which contributes to neuroinflammation and cell death 66, 67. The genes *Mb21d1* and *Tmem173* encode two key proteins in the cGAS/STING pathway of the innate immune response, which has been shown to be activated in the microglia of the spinal cord after sciatic nerve injury 68. Furthermore, several genes (*Apoe*, *Blnk*, and autophagy-related genes *Lamp1*, *Pink1*) were found to be downregulated in SCI/*Becn1^+/-^* mice compared to SCI/WT animals. Apoe (Apolipoprotein E) plays an important role in lipid transport in the CNS, regulating neuron survival and sprouting and is also involved in innate and adaptive immune responses, controlling for instance the survival of myeloid-derived suppressor cells 69-71. Recent studies showed that *Apoe* deficiency impaired neuronal repair and nerve regeneration after SCI 72, 73. An important regulator in adaptive immune response *Blnk* (B Cell Linker) is a cytoplasmic linker and adaptor protein that plays a critical role in B cell development 74, 75. Although usually associated with the antibody producing B cells, *Blnk* has also been found to be significantly upregulated in a mouse model of Alzheimer's disease following exposure to Aβ 76. In addition, the autophagy-related gene *Pink1* is involved with mitophagy, the down-regulation of which would indicate inhibited clearance of dysfunction mitochondria, leading to the increase of oxidative stress and inflammation 77. Taken together, genetically inhibiting autophagy exacerbates post-injury neuroinflammation at the transcriptomic level.

Autophagy has been shown to play a major role in determination of pro- and anti- inflammatory profiles in other inflammatory models 78, 79. In SCI, while decreased levels of autophagy in *Becn1^+/-^* mice did not affect expression levels of anti-inflammatory cytokines, we observed significant increase in several pro-inflammatory cytokines in SCI/*Becn1^+/-^* than SCI/WT, indicating a more exaggerated immune response. Flow cytometry analysis further confirmed marked increase of pro-inflammatory cytokines TNF and IL-1β in microglia derived from *Becn1^+/-^* mice compared to their WT counterparts. More recently, autophagy has been shown to control cellular innate immunity including the inflammasome by directly targeting its components for lysosomal degradation 80. In the present study, we observed increased protein expression of NLRP3 and the innate immunity pathways in SCI/*Becn1^+/-^* mice, coinciding with accumulation of autophagosome and increased expression of microglia/macrophages marker IBA-1, suggesting exacerbation of innate immune responses following SCI.

The function of autophagy in SCI remained controversial for a long time, with both beneficial and detrimental roles. However, numerous experimental observations including ours indicate that persistent neuroinflammation contributes to long-term neurological functional deficits. Early potentiation of neuroinflammation and innate immune responses in injured mice with autophagy deficiency was associated with poor functional recovery and exacerbated tissue damage for up to 6 weeks after injury. However, in addition to modulation of inflammatory responses, autophagy is important for neuronal survival and function 1, 4, 5. The confounding effects of using global autophagy hypomorph *Becn1^+/-^* mice are perturbed autophagy in all cell types. Cell type specific approach will be necessary for future investigation, as inhibition of autophagy in neurons and oligodendrocytes is detrimental. Since sex differences in acute neuroinflammation and neurological outcome have been widely established in experimental models of SCI 81, 82, it will also be necessary to perform similar studies in female mice to determine if sex may affect the role of autophagy in SCI.

Since inhibition of autophagy contributes to both SCI-induced inflammation and neuronal cell death, enhancement of autophagy may represent an attractive treatment option. Pharmaceutical treatments known to enhance levels of autophagy have been reported to improve SCI outcomes. However, the drugs used have multiple functions and it is not known if their effects on SCI were mediated directly via autophagy. For example, the autophagy enhancer Rapamycin has been shown in many studies to be protective after SCI 6, 22-24. However, contrasting results have also been reported 83, such as exacerbation of cardiovascular complications caused by SCI 84. In addition, inhibition of mTOR also affects protein synthesis and immune and inflammatory responses 23, 25-27, which could lead to autophagy independent augmentation of SCI recovery. The disaccharide trehalose, being an autophagy enhancer independent of mTOR, is a viable alternative for therapeutic purposes. Known to enhance lysosomal biogenesis by promoting activity of the transcription factor EB (TFEB), trehalose has been shown to be effective in alleviating neurological deficits for multiple rodent models of neurodegeneration 28, 29, 85 and in a rabbit model of spinal cord ischemia 30. However, the precise mechanisms through which trehalose acts on the autophagy pathway have not been established. We found that trehalose increases autophagy flux which is impaired in the acute phase of injury, accompanied by reduction of innate immune responses and inflammation following SCI. Thus, trehalose may exert important effects on modulation of the inflammasome and the innate immunity pathways, resulting in better functional outcome following SCI. However, we cannot rule out the effects of long-term trehalose treatment on lysosome permeabilization in the present model. Trehalose is known to impose a low-grade lysosomal stress, resulting in the activation of TFEB-dependent lysosome biogenesis 86. Moreover, our recent study 87 in a mouse traumatic brain injury (TBI) model demonstrated chronic increases of lysosomal enzyme activity in microglia derived from long-term (9 weeks) trehalose-treated mice that were indicative of improved autophagy function and associated with better functional outcome following TBI. Whether trehalose acts directly on the microglia or in the periphery to achieve this effect is not clear.

## Conclusion

**Figure 12:**
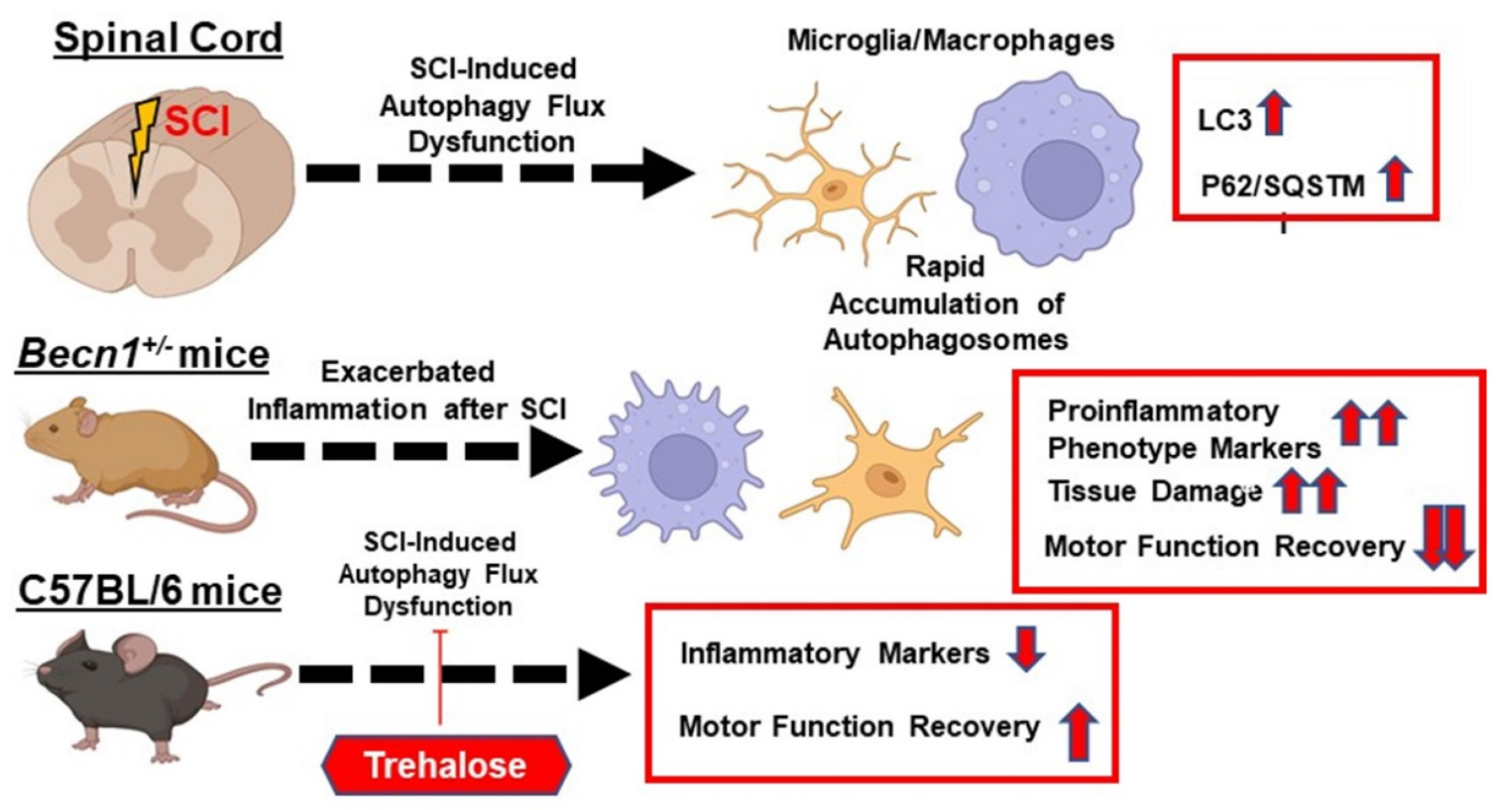
** The function of autophagy in modulating neuroinflammation following spinal cord injury (SCI).** The function of autophagy in modulating neuroinflammation following spinal cord injury (SCI). SCI impairs autophagic flux in microglia/macrophages. Genetically or pharmacologically manipulating autophagy can modulate SCI-mediated pro-inflammatory response and neurological recovery.

Taken together, our transcriptome profile, histological, cellular, and molecular findings provided complementary evidence that autophagy deficiency exacerbated neuroinflammation following SCI (**Figure 12**). We showed that genetically or pharmacologically manipulating autophagy can modulate SCI-mediated pro-inflammatory response and neurological recovery.

## Supplementary Material

Supplementary materials, figures.

## Abbreviations

Beclin-1 (Becn1): coiled-coil myosin-like BCL2-interacting protein
BMS: Basso mouse scale
cGAS: cyclic GMP-AMP synthase
CNS: central nervous system
CX3CR1-GFP: C-X3-C motif chemokine receptor 1-green fluorescent protein
FACS: fluorescence-activated cell sorting
IACUC: Institutional Animal Care and Use Committee
IBA-1: ionized calcium binding adaptor molecule 1
IHC: immunohistochemistry
LBS: ladder beam score
LC3: microtubule-associated protein 1A/1B light chain 3
LFB: Luxol fast blue
mTOR: mammalian target of rapamycin
NLRP3: NOD-, LRR- and pyrin domain-containing protein 3
p62/SQSTM1: ubiquitin-binding protein p62/ Sequestosome-1
PI3K: phosphatidylinositol-3 kinase
qPCR: quantitative polymerase chain reaction
STING: stimulator of interferon genes
SWM: spared white matter
SCI: spinal cord injury
WT: wildtype

## References

[1] Wu J, Lipinski MM (2019). Autophagy in Neurotrauma: Good, Bad, or Dysregulated. Cells.

[2] Kanno H, Ozawa H, Sekiguchi A, Itoi E (2009). The role of autophagy in spinal cord injury. Autophagy.

[3] Liu S, Sarkar C, Dinizo M, Faden AI, Koh EY, Lipinski MM (2015). Disrupted autophagy after spinal cord injury is associated with ER stress and neuronal cell death. Cell Death Dis.

[4] Liu S, Li Y, Choi HMC, Sarkar C, Koh EY, Wu J (2018). Lysosomal damage after spinal cord injury causes accumulation of RIPK1 and RIPK3 proteins and potentiation of necroptosis. Cell Death Dis.

[5] Li Y, Jones JW, H MCC, Sarkar C, Kane MA, Koh EY (2019). cPLA2 activation contributes to lysosomal defects leading to impairment of autophagy after spinal cord injury. Cell Death Dis.

[6] Sekiguchi A, Kanno H, Ozawa H, Yamaya S, Itoi E (2012). Rapamycin promotes autophagy and reduces neural tissue damage and locomotor impairment after spinal cord injury in mice. J Neurotrauma.

[7] Zhang HY, Wang ZG, Wu FZ, Kong XX, Yang J, Lin BB (2013). Regulation of autophagy and ubiquitinated protein accumulation by bFGF promotes functional recovery and neural protection in a rat model of spinal cord injury. Mol Neurobiol.

[8] Cadwell K, Liu JY, Brown SL, Miyoshi H, Loh J, Lennerz JK (2008). A key role for autophagy and the autophagy gene Atg16l1 in mouse and human intestinal Paneth cells. Nature.

[9] Sil S, Niu F, Tom E, Liao K, Periyasamy P, Buch S (2019). Cocaine Mediated Neuroinflammation: Role of Dysregulated Autophagy in Pericytes. Mol Neurobiol.

[10] Cheng J, Liao Y, Dong Y, Hu H, Yang N, Kong X (2020). Microglial autophagy defect causes parkinson disease-like symptoms by accelerating inflammasome activation in mice. Autophagy.

[11] Choi I, Zhang Y, Seegobin SP, Pruvost M, Wang Q, Purtell K (2020). Microglia clear neuron-released alpha-synuclein via selective autophagy and prevent neurodegeneration. Nat Commun.

[12] Shao BZ, Ke P, Xu ZQ, Wei W, Cheng MH, Han BZ (2017). Autophagy Plays an Important Role in Anti-inflammatory Mechanisms Stimulated by Alpha7 Nicotinic Acetylcholine Receptor. Front Immunol.

[13] Kumar A, Kalita J, Sinha RA, Singh G, B A, Shukla M (2020). Impaired Autophagy Flux is Associated with Proinflammatory Microglia Activation Following Japanese Encephalitis Virus Infection. Neurochem Res.

[14] Heckmann BL, Teubner BJW, Boada-Romero E, Tummers B, Guy C, Fitzgerald P (2020). Noncanonical function of an autophagy protein prevents spontaneous Alzheimer's disease. Sci Adv.

[15] Espinosa-Garcia C, Atif F, Yousuf S, Sayeed I, Neigh GN, Stein DG (2020). Progesterone Attenuates Stress-Induced NLRP3 Inflammasome Activation and Enhances Autophagy following Ischemic Brain Injury. Int J Mol Sci.

[16] Hill SM, Wrobel L, Rubinsztein DC (2019). Post-translational modifications of Beclin 1 provide multiple strategies for autophagy regulation. Cell Death Differ.

[17] Sun T, Li X, Zhang P, Chen WD, Zhang HL, Li DD (2015). Acetylation of Beclin 1 inhibits autophagosome maturation and promotes tumour growth. Nat Commun.

[18] Fekadu J, Rami A (2016). Beclin-1 Deficiency Alters Autophagosome Formation, Lysosome Biogenesis and Enhances Neuronal Vulnerability of HT22 Hippocampal Cells. Mol Neurobiol.

[19] Gladue DP, O'Donnell V, Baker-Branstetter R, Holinka LG, Pacheco JM, Fernandez-Sainz I (2012). Foot-and-mouth disease virus nonstructural protein 2C interacts with Beclin1, modulating virus replication. J Virol.

[20] Qu X, Yu J, Bhagat G, Furuya N, Hibshoosh H, Troxel A (2003). Promotion of tumorigenesis by heterozygous disruption of the beclin 1 autophagy gene. J Clin Invest.

[21] Zhang D, Zhu D, Wang F, Zhu JC, Zhai X, Yuan Y (2020). Therapeutic effect of regulating autophagy in spinal cord injury: a network meta-analysis of direct and indirect comparisons. Neural Regen Res.

[22] Cai Z, Yan LJ (2013). Rapamycin, Autophagy, and Alzheimer's Disease. J Biochem Pharmacol Res.

[23] Goldshmit Y, Kanner S, Zacs M, Frisca F, Pinto AR, Currie PD (2015). Rapamycin increases neuronal survival, reduces inflammation and astrocyte proliferation after spinal cord injury. Mol Cell Neurosci.

[24] Tang P, Hou H, Zhang L, Lan X, Mao Z, Liu D (2014). Autophagy reduces neuronal damage and promotes locomotor recovery via inhibition of apoptosis after spinal cord injury in rats. Mol Neurobiol.

[25] Vargova I, Machova Urdzikova L, Karova K, Smejkalova B, Sursal T, Cimermanova V (2021). Involvement of mTOR Pathways in Recovery from Spinal Cord Injury by Modulation of Autophagy and Immune Response. Biomedicines.

[26] Du K, Zheng S, Zhang Q, Li S, Gao X, Wang J (2015). Pten Deletion Promotes Regrowth of Corticospinal Tract Axons 1 Year after Spinal Cord Injury. J Neurosci.

[27] Lu P, Wang Y, Graham L, McHale K, Gao M, Wu D (2012). Long-distance growth and connectivity of neural stem cells after severe spinal cord injury. Cell.

[28] Schaeffer V, Lavenir I, Ozcelik S, Tolnay M, Winkler DT, Goedert M (2012). Stimulation of autophagy reduces neurodegeneration in a mouse model of human tauopathy. Brain.

[29] Zhang X, Chen S, Song L, Tang Y, Shen Y, Jia L (2014). MTOR-independent, autophagic enhancer trehalose prolongs motor neuron survival and ameliorates the autophagic flux defect in a mouse model of amyotrophic lateral sclerosis. Autophagy.

[30] Takahashi S, Isaka M, Hamaishi M, Imai K, Orihashi K, Sueda T (2014). Trehalose protects against spinal cord ischemia in rabbits. J Vasc Surg.

[31] Li Y, Ritzel RM, He J, Cao T, Sabirzhanov B, Li H (2021). The voltage-gated proton channel Hv1 plays a detrimental role in contusion spinal cord injury via extracellular acidosis-mediated neuroinflammation. Brain Behav Immun.

[32] Xu C, Li X, Wang F, Weng H, Yang P (2013). Trehalose prevents neural tube defects by correcting maternal diabetes-suppressed autophagy and neurogenesis. Am J Physiol Endocrinol Metab.

[33] Portbury SD, Hare DJ, Finkelstein DI, Adlard PA (2017). Trehalose improves traumatic brain injury-induced cognitive impairment. PLoS One.

[34] Portbury SD, Hare DJ, Sgambelloni C, Perronnes K, Portbury AJ, Finkelstein DI (2017). Trehalose Improves Cognition in the Transgenic Tg2576 Mouse Model of Alzheimer's Disease. J Alzheimers Dis.

[35] Basso DM, Fisher LC, Anderson AJ, Jakeman LB, McTigue DM, Popovich PG (2006). Basso Mouse Scale for locomotion detects differences in recovery after spinal cord injury in five common mouse strains. J Neurotrauma.

[36] Sabirzhanov B, Li Y, Coll-Miro M, Matyas JJ, He J, Kumar A (2019). Inhibition of NOX2 signaling limits pain-related behavior and improves motor function in male mice after spinal cord injury: Participation of IL-10/miR-155 pathways. Brain Behav Immun.

[37] Cummings BJ, Engesser-Cesar C, Cadena G, Anderson AJ (2007). Adaptation of a ladder beam walking task to assess locomotor recovery in mice following spinal cord injury. Behav Brain Res.

[38] Ritzel RM, He J, Li Y, Cao T, Khan N, Shim B (2021). Proton extrusion during oxidative burst in microglia exacerbates pathological acidosis following traumatic brain injury. Glia.

[39] Ritzel RM, Patel AR, Grenier JM, Crapser J, Verma R, Jellison ER (2015). Functional differences between microglia and monocytes after ischemic stroke. J Neuroinflammation.

[40] Ritzel RM, Li Y, He J, Khan N, Doran SJ, Faden AI (2020). Sustained neuronal and microglial alterations are associated with diverse neurobehavioral dysfunction long after experimental brain injury. Neurobiol Dis.

[41] Li Y, Ritzel RM, Khan N, Cao T, He J, Lei Z (2020). Delayed microglial depletion after spinal cord injury reduces chronic inflammation and neurodegeneration in the brain and improves neurological recovery in male mice. Theranostics.

[42] Vandesompele J, De Preter K, Pattyn F, Poppe B, Van Roy N, De Paepe A (2002). Accurate normalization of real-time quantitative RT-PCR data by geometric averaging of multiple internal control genes. Genome Biol.

[43] Chen EY, Tan CM, Kou Y, Duan Q, Wang Z, Meirelles GV (2013). Enrichr: interactive and collaborative HTML5 gene list enrichment analysis tool. BMC Bioinformatics.

[44] Kuleshov MV, Jones MR, Rouillard AD, Fernandez NF, Duan Q, Wang Z (2016). Enrichr: a comprehensive gene set enrichment analysis web server 2016 update. Nucleic Acids Res.

[45] Xie Z, Bailey A, Kuleshov MV, Clarke DJB, Evangelista JE, Jenkins SL (2021). Gene Set Knowledge Discovery with Enrichr. Curr Protoc.

[46] Wang H, Horbinski C, Wu H, Liu Y, Sheng S, Liu J (2016). NanoStringDiff: a novel statistical method for differential expression analysis based on NanoString nCounter data. Nucleic Acids Res.

[47] Jung S, Aliberti J, Graemmel P, Sunshine MJ, Kreutzberg GW, Sher A (2000). Analysis of fractalkine receptor CX(3)CR1 function by targeted deletion and green fluorescent protein reporter gene insertion. Mol Cell Biol.

[48] Jha MK, Lee S, Park DH, Kook H, Park KG, Lee IK (2015). Diverse functional roles of lipocalin-2 in the central nervous system. Neurosci Biobehav Rev.

[49] Jeon S, Jha MK, Ock J, Seo J, Jin M, Cho H (2013). Role of lipocalin-2-chemokine axis in the development of neuropathic pain following peripheral nerve injury. J Biol Chem.

[50] Rathore KI, Berard JL, Redensek A, Chierzi S, Lopez-Vales R, Santos M (2011). Lipocalin 2 plays an immunomodulatory role and has detrimental effects after spinal cord injury. J Neurosci.

[51] Thompson WL, Van Eldik LJ (2009). Inflammatory cytokines stimulate the chemokines CCL2/MCP-1 and CCL7/MCP-3 through NFkB and MAPK dependent pathways in rat astrocytes [corrected]. Brain Res.

[52] Rong Y, Ji C, Wang Z, Ge X, Wang J, Ye W (2021). Small extracellular vesicles encapsulating CCL2 from activated astrocytes induce microglial activation and neuronal apoptosis after traumatic spinal cord injury. J Neuroinflammation.

[53] Xu P, Zhang F, Chang MM, Zhong C, Sun CH, Zhu HR (2021). Recruitment of gammadelta T cells to the lesion via the CCL2/CCR2 signaling after spinal cord injury. J Neuroinflammation.

[54] Kwiatkowski K, Popiolek-Barczyk K, Piotrowska A, Rojewska E, Ciapala K, Makuch W (2019). Chemokines CCL2 and CCL7, but not CCL12, play a significant role in the development of pain-related behavior and opioid-induced analgesia. Cytokine.

[55] Mordillo-Mateos L, Sanchez-Ramos A, Coperchini F, Bustos-Guadamillas I, Alonso-Bonilla C, Vargas-Baquero E (2019). Development of chronic pain in males with traumatic spinal cord injury: role of circulating levels of the chemokines CCL2 and CXCL10 in subacute stage. Spinal Cord.

[56] Haque M, Siegel RJ, Fox DA, Ahmed S (2021). Interferon-stimulated GTPases in autoimmune and inflammatory diseases: promising role for the guanylate-binding protein (GBP) family. Rheumatology (Oxford).

[57] Yu P, Li Y, Li Y, Miao Z, Peppelenbosch MP, Pan Q (2020). Guanylate-binding protein 2 orchestrates innate immune responses against murine norovirus and is antagonized by the viral protein NS7. J Biol Chem.

[58] Johns CE, Galam L (2022). Guanylate Binding Protein 1 (GBP1): A Key Protein in Inflammatory Pyroptosis. Cell Biochem Biophys.

[59] Sahin B, Albayrak BS, Ismailoglu O, Gorgulu A (2011). The effects of medroxy progesterone acetate on the pro-inflammatory cytokines, TNF-alpha and IL-1beta in the early phase of the spinal cord injury. Neurol Res.

[60] Lin WP, Lin JH, Cai B, Shi JX, Li WJ, Choudhury GR (2016). Effect of adenovirus-mediated RNA interference of IL-1beta expression on spinal cord injury in rats. Spinal Cord.

[61] Zhou W, Yuan T, Gao Y, Yin P, Liu W, Pan C (2017). IL-1beta-induces NF-kappaB and upregulates microRNA-372 to inhibit spinal cord injury recovery. J Neurophysiol.

[62] Nakamura M, Okada S, Toyama Y, Okano H (2005). Role of IL-6 in spinal cord injury in a mouse model. Clin Rev Allergy Immunol.

[63] Niu C, Yip HK (2011). Neuroprotective signaling mechanisms of telomerase are regulated by brain-derived neurotrophic factor in rat spinal cord motor neurons. J Neuropathol Exp Neurol.

[64] Kanda H, Kobayashi K, Yamanaka H, Okubo M, Noguchi K (2017). Microglial TNFalpha Induces COX2 and PGI2 Synthase Expression in Spinal Endothelial Cells during Neuropathic Pain. eNeuro.

[65] Ji H, Zhang Y, Chen C, Li H, He B, Yang T (2021). D-dopachrome tautomerase activates COX2/PGE2 pathway of astrocytes to mediate inflammation following spinal cord injury. J Neuroinflammation.

[66] Huang JH, Fu CH, Xu Y, Yin XM, Cao Y, Lin FY (2020). Extracellular Vesicles Derived from Epidural Fat-Mesenchymal Stem Cells Attenuate NLRP3 Inflammasome Activation and Improve Functional Recovery After Spinal Cord Injury. Neurochem Res.

[67] Zhang M, Wang L, Huang S, He X (2020). MicroRNA-223 targets NLRP3 to relieve inflammation and alleviate spinal cord injury. Life Sci.

[68] Morozzi G, Rothen J, Toussaint G, De Lange K, Westritschnig K, Doelemeyer A (2021). STING regulates peripheral nerve regeneration and colony stimulating factor 1 receptor (CSF1R) processing in microglia. iScience.

[69] Huang Y, Mahley RW (2014). Apolipoprotein E: structure and function in lipid metabolism, neurobiology, and Alzheimer's diseases. Neurobiol Dis.

[70] Fagan AM, Bu G, Sun Y, Daugherty A, Holtzman DM (1996). Apolipoprotein E-containing high density lipoprotein promotes neurite outgrowth and is a ligand for the low density lipoprotein receptor-related protein. J Biol Chem.

[71] Yamamoto K, Yamada H, Wakana N, Kikai M, Terada K, Wada N (2018). Augmented neutrophil extracellular traps formation promotes atherosclerosis development in socially defeated apoE(-/-) mice. Biochem Biophys Res Commun.

[72] Yang X, Chen S, Shao Z, Li Y, Wu H, Li X (2018). Apolipoprotein E Deficiency Exacerbates Spinal Cord Injury in Mice: Inflammatory Response and Oxidative Stress Mediated by NF-kappaB Signaling Pathway. Front Cell Neurosci.

[73] Cheng X, Zheng Y, Bu P, Qi X, Fan C, Li F (2018). Apolipoprotein E as a novel therapeutic neuroprotection target after traumatic spinal cord injury. Exp Neurol.

[74] Taguchi T, Kiyokawa N, Takenouch H, Matsui J, Tang WR, Nakajima H (2004). Deficiency of BLNK hampers PLC-gamma2 phosphorylation and Ca2+ influx induced by the pre-B-cell receptor in human pre-B cells. Immunology.

[75] Draber P, Vonkova I, Stepanek O, Hrdinka M, Kucova M, Skopcova T (2011). SCIMP, a transmembrane adaptor protein involved in major histocompatibility complex class II signaling. Mol Cell Biol.

[76] Sierksma A, Lu A, Mancuso R, Fattorelli N, Thrupp N, Salta E (2020). Novel Alzheimer risk genes determine the microglia response to amyloid-beta but not to TAU pathology. EMBO Mol Med.

[77] Gu C, Li L, Huang Y, Qian D, Liu W, Zhang C (2020). Salidroside Ameliorates Mitochondria-Dependent Neuronal Apoptosis after Spinal Cord Ischemia-Reperfusion Injury Partially through Inhibiting Oxidative Stress and Promoting Mitophagy. Oxid Med Cell Longev.

[78] Zhang M, Liu F, Zhou P, Wang Q, Xu C, Li Y (2019). The MTOR signaling pathway regulates macrophage differentiation from mouse myeloid progenitors by inhibiting autophagy. Autophagy.

[79] Ma K, Guo J, Wang G, Ni Q, Liu X (2020). Toll-Like Receptor 2-Mediated Autophagy Promotes Microglial Cell Death by Modulating the Microglial M1/M2 Phenotype. Inflammation.

[80] Shi CS, Shenderov K, Huang NN, Kabat J, Abu-Asab M, Fitzgerald KA (2012). Activation of autophagy by inflammatory signals limits IL-1beta production by targeting ubiquitinated inflammasomes for destruction. Nat Immunol.

[81] Stewart AN, Lowe JL, Glaser EP, Mott CA, Shahidehpour RK, McFarlane KE (2021). Acute inflammatory profiles differ with sex and age after spinal cord injury. J Neuroinflammation.

[82] Li Y, Ritzel RM, Lei Z, Cao T, He J, Faden AI (2021). Sexual dimorphism in neurological function after SCI is associated with disrupted neuroinflammation in both injured spinal cord and brain. Brain Behav Immun.

[83] Saraswat Ohri S, Bankston AN, Mullins SA, Liu Y, Andres KR, Beare JE (2018). Blocking Autophagy in Oligodendrocytes Limits Functional Recovery after Spinal Cord Injury. J Neurosci.

[84] Eldahan KC, Cox DH, Gollihue JL, Patel SP, Rabchevsky AG (2018). Rapamycin Exacerbates Cardiovascular Dysfunction after Complete High-Thoracic Spinal Cord Injury. J Neurotrauma.

[85] Rusmini P, Cortese K, Crippa V, Cristofani R, Cicardi ME, Ferrari V (2019). Trehalose induces autophagy via lysosomal-mediated TFEB activation in models of motoneuron degeneration. Autophagy.

[86] Jeong SJ, Stitham J, Evans TD, Zhang X, Rodriguez-Velez A, Yeh YS (2021). Trehalose causes low-grade lysosomal stress to activate TFEB and the autophagy-lysosome biogenesis response. Autophagy.

[87] Ritzel RM, Li Y, Lei Z, Carter J, He J, Choi HMC (2022). Functional and transcriptional profiling of microglial activation during the chronic phase of TBI identifies an age-related driver of poor outcome in old mice. Geroscience.

