# Supplementary material for: Impairment of autophagy after spinal cord injury potentiates neuroinflammation and motor function deficit in mice
Source: Theranostics. 2022 Jul 11;12(12):5364–88. doi: 10.7150/thno.72713 (PMC9330534; doi:10.7150/thno.72713)
Supplement: Supplementary file 1 — Supplementary materials, figures. [file thnov12p5364s1.pdf]

Impairment of autophagy after spinal cord injury potentiates neuroinflammation and motor function deficit in mice

Yun Li*, Zhuofan Lei*, Rodney M. Ritzel, Junyun He, Hui Li, Harry M. C. Choi, Marta M. Lipinski[#], Junfang Wu[#]

Department of Anesthesiology and Center for Shock, Trauma and Anesthesiology Research (STAR), University of Maryland School of Medicine, Baltimore, MD, 21201 USA.

Supplementary Material
Supplementary figures 1-7.
Supplementary Figure legends

Supplementary Material

Open field (OF) test [40,41]: Spontaneous locomotor activity was examined by OF test. Mice were placed in a corner faced towards the wall of the OF apparatus (22.5 × 22.5 cm) and allowed to freely explore the chamber for 5 min. Six parameters, including total distance travelled (m), mean speed (m/s), max speed (m/s), time spent in the outside/inside zone (s) and time immobile (s) were recorded by the computer-based ANY-maze automated videotracking system (Stoelting).

Y-maze test [40,41]: The Y-maze (Stoelting) was built of black plastic and consisted of three identical arms set an angle of 120◦ from each other. Each arm measured at 38.1 cm long, 5 cm wide, and 12.7 cm high. Before the test, one arm was randomly selected as the “starting” position. The mouse was placed into the end of the “start” arm and allowed to explore the maze freely for 6 min. The arm entries (arms A–C) were recorded with the ANY-maze videotracking software. An entry by the mouse was defined as all four paws being inside of the arm. An alternation was completed when the mouse entered three different arms in consecutive order. The percentage of alternation in a test was calculated with the following equation: total alternations ×100/(total arm entries − 2). If a mouse scored significantly above 50% alternations (the chance level for choosing the unfamiliar arm), this was indicative of the mouse having functional working memory.

Novel object recognition (NOR) test [40,41]: The mice were habituated in the OF chamber for 5 min prior to the first test day. After 24 h, mice were placed into the same OF chamber with two identical objects placed near the left and right corners. During the sample phase, mice were allowed to explore the chamber freely under dim light until they spent a total of 30 s exploring the objects (exploration time was recorded with a stopwatch when the front paws or nose contacted the object). Mice were then returned to their home cages. After 24 h, during the choice phase, object recognition was tested by substituting a familiar object with a novel object (object location was counterbalanced across mice). Time spent with each object was recorded with a stopwatch. Because mice inherently prefer to explore novel objects, mice spending more time than the chance level (15 s) with the novel object indicates intact memory for the familiar object and a normal learning memory function.

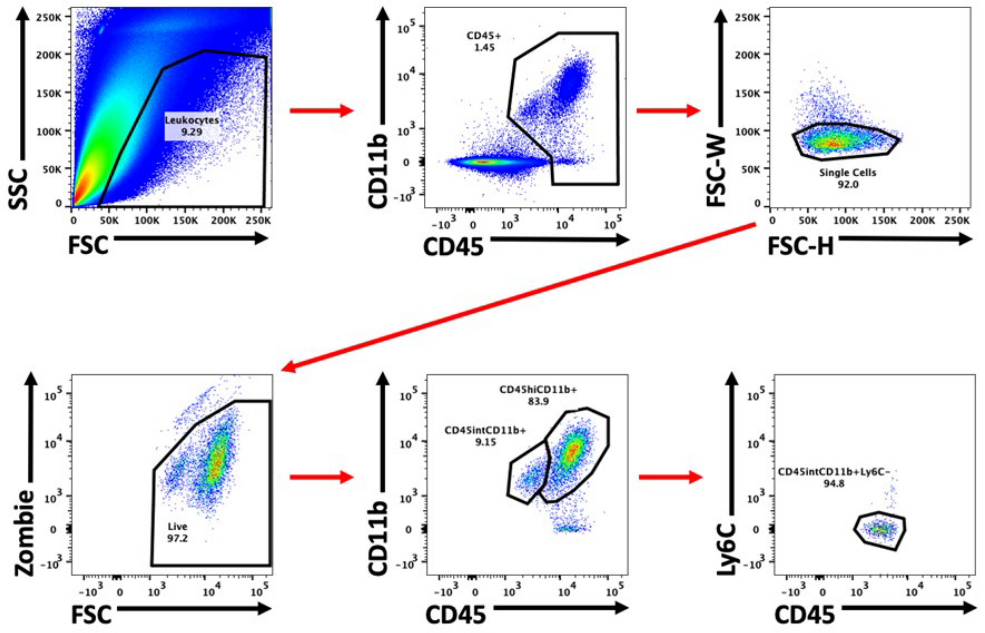

Figure S1. Gating strategy for immune cells in the injured spinal cord. A first leukocyte gate was defined in the dot plot Forward Scatter (SSC) versus Forward Scatter (FSC) using a splenocyte reference. A second gate (CD11b versus CD45) was defined to isolate all CD45-positive immune cells. A third gate was used to discriminate single cells from doublets (FSC-W versus FSC-H). A fourth gate was created to identify living cells (Zombie versus FSC). A fifth gate (CD11b versus CD45) was created to identify all CD45 populations (i.e., $CD45^{int}CD11b^{+}$ microglia and $CD45^{hi}CD11b^{+}$ myeloid cells). A sixth gate was created to discriminate any microglia from peripheral borne myeloid cells (Ly6C versus CD45).

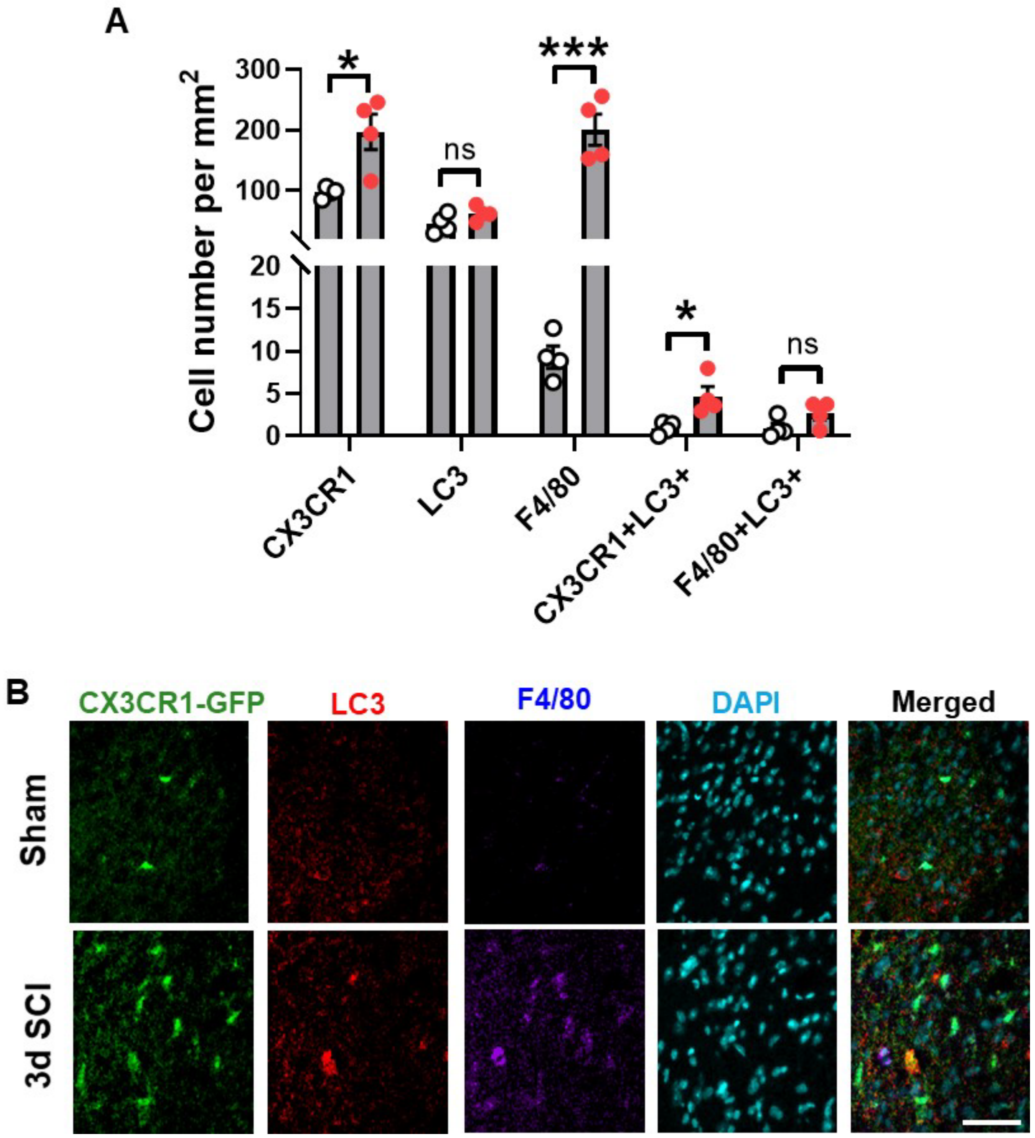

Figure S2. Autophagosomes acutely accumulate in activated microglia and infiltrating macrophages at 3 days after SCI. Young adult male *CX3CR1-GFP* mice underwent moderate contusion injury at T10. (**A-B**) Representative images and cell count quantification of LC3 (red) and F4/80 (blue) positive cells. n = 4 mice/group. *$p < 0.05$, **$p < 0.01$, ***$p < 0.001$. Two-tailed unpaired t-test. Scale bar = 50 μm.

A

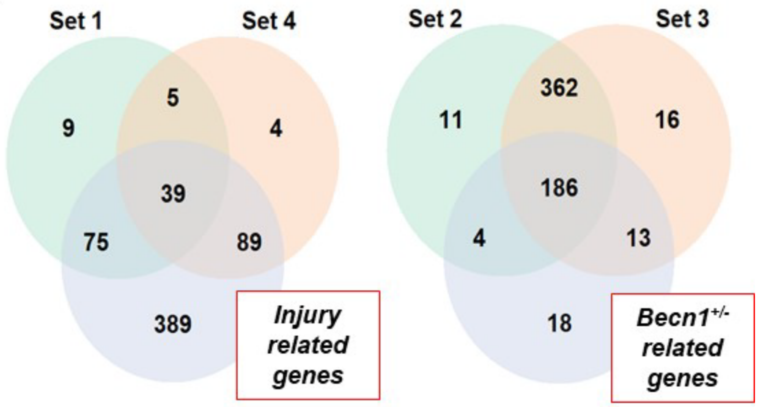

B

Pairwise Comparison	DE Genes ↓ Expression	DE Genes ↑ Expression
1	35	93
2	113	450
3	133	444
4	61	76

Figure S3. Autophagy deficiency alters neuroinflammation transcriptome within the spinal cord after acute injury. (**A**) Venn diagram demonstrates the separation of total injury genes into those modified by *Becn1*$^{+/-}$ or not. (**B**) Differential expression (DE) analysis was performed on pairwise group comparisons using the nSolver Analysis Software. Genes upregulated or downregulated within each set of pairwise comparison are presented as lists. n = 5 mice/group. Set 1: Sham/*Becn1*$^{+/-}$ vs. Sham/WT –Comparison 1; Set 2: SCI/WT vs. Sham/WT -Comparison 2; Set 3: SCI/ *Becn1*$^{+/-}$ vs. Sham/*Becn1*$^{+/-}$ – Comparison 3; and Set 4: SCI/ *Becn1*$^{+/-}$ vs. SCI/WT – Comparison 4.

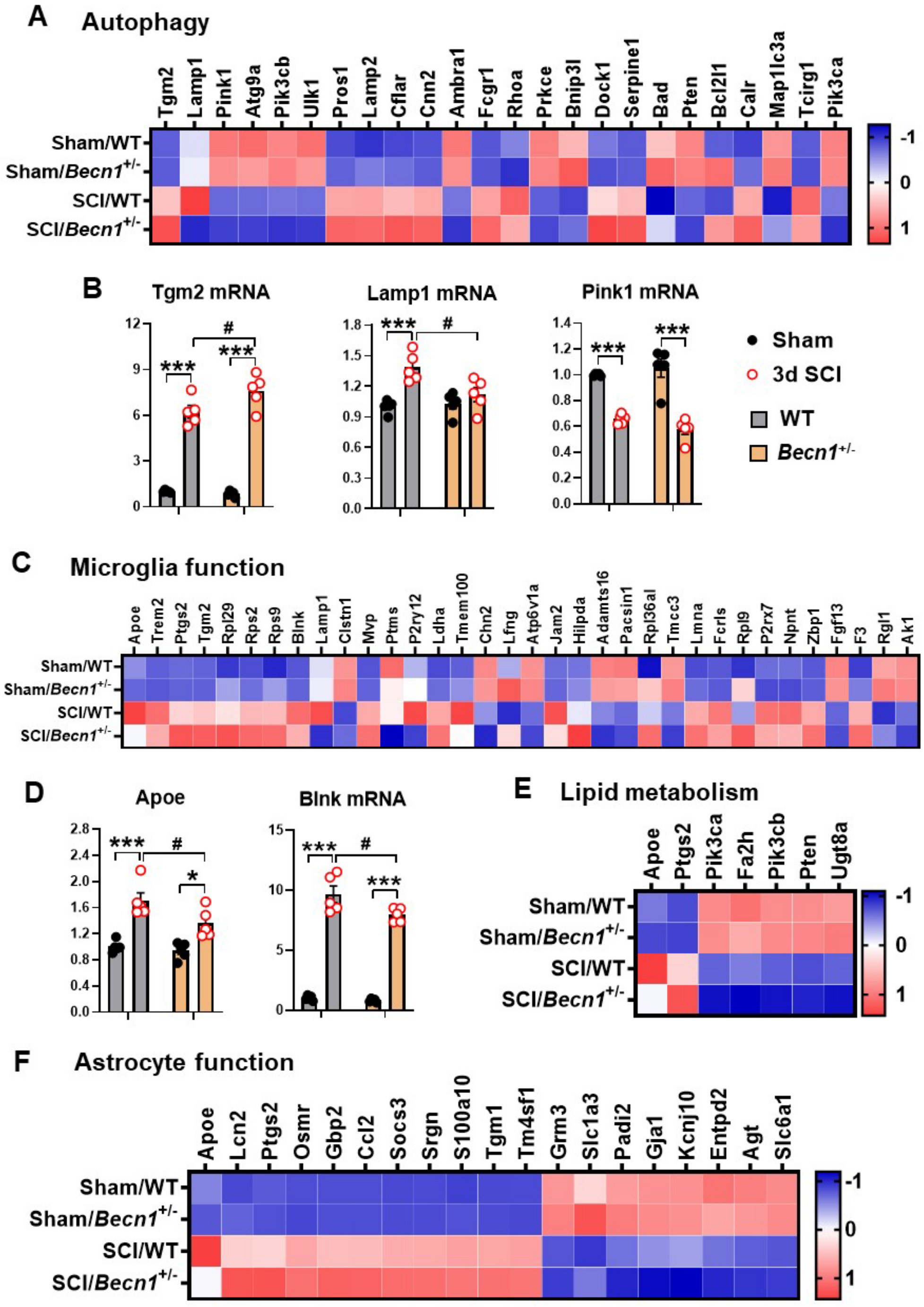

Figure S4. Autophagy deficiency leads to high transcription of genes related to autophagy, microglia function, lipid metabolism, and astrocyte function. Analysis of DE genes between SCI/*Becn1*$^{+/-}$ and SCI/WT show key genes related to Autophagy signaling (**A**) and qPCR validation (**B**), Microglia function (**C-D**), Lipid metabolism (**E**), and Astrocyte function (**F**) being modified by autophagy deficiency. All transcriptome data have been normalized to z-scores for heatmaps and linear fold change to Sham/WT were used for column graph with bars. n = 5 mice/group. **p* < 0.05, ****p* < 0.001 vs. Sham groups; $^{\#}p$ < 0.05 vs. SCI/WT. Two-way ANOVA followed by Tukey's multiple comparison.

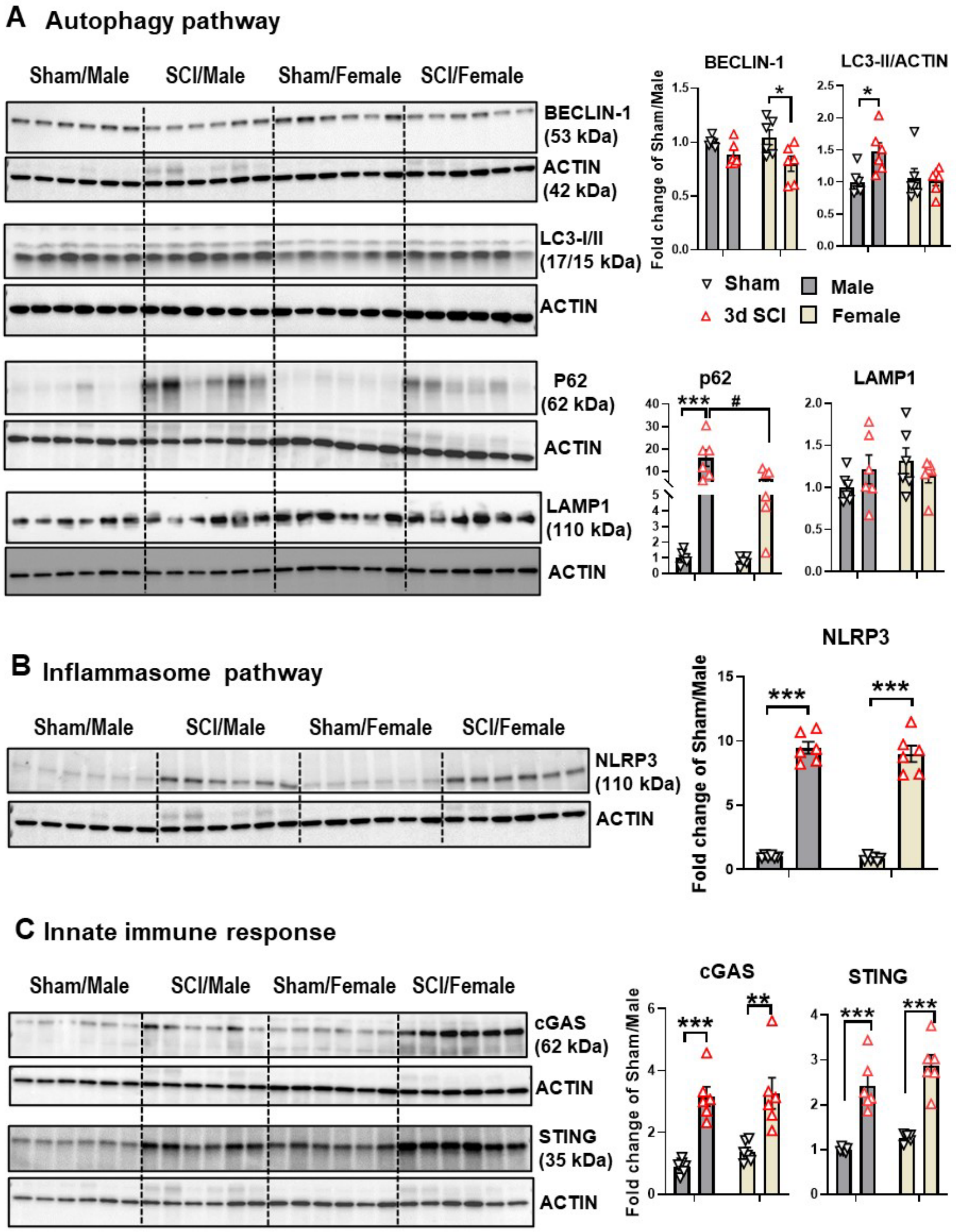

Figure S5. Effects of sex on the protein expression levels of key inflammatory and autophagic markers at 3 d post-injury. Young adult C57BL/6 mice were subjected to moderate contusion injury and Western blotting was used to examine autophagic and inflammatory markers at 3 d post-injury. (**A**) Expression of BECLIN-1, LC3-II, p62 and LAMP1. (**B**) Expression of inflammasomes NLRP3. (**C**) Expression of the markers cGAS and STING for innate immune response following SCI. n = 6 mice/group, * $p < 0.05$, ** $p < 0.01$, *** $p < 0.001$ vs. Sham groups. # $p < 0.05$ vs. SCI/Male. Two-way ANOVA followed by Tukey's multiple comparison.

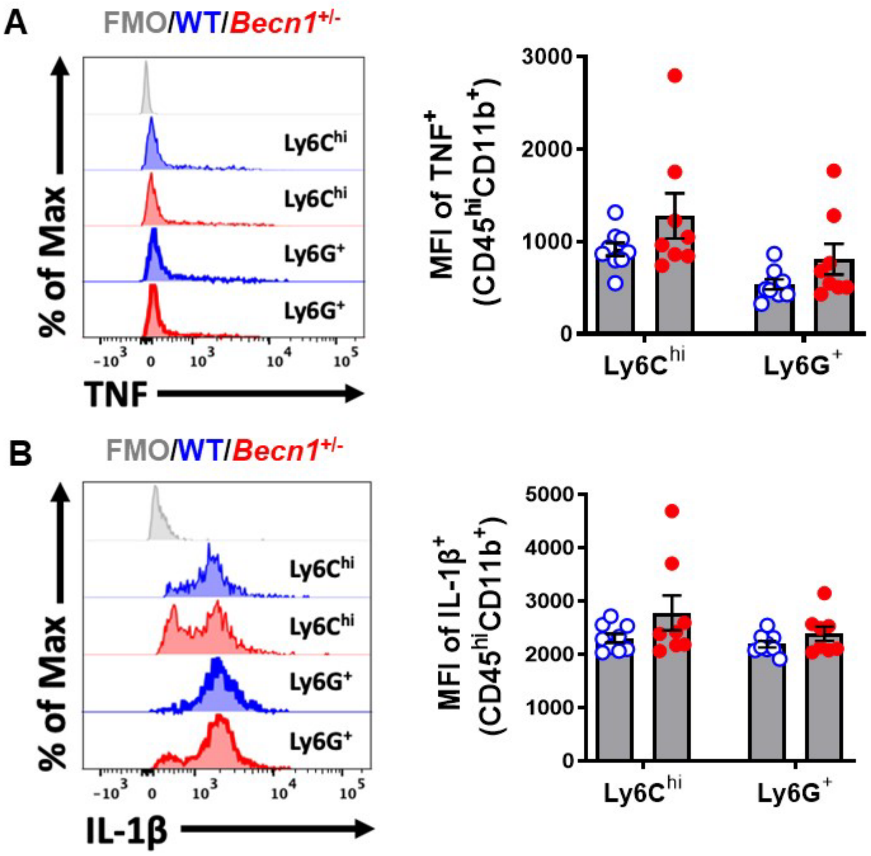

Figure S6. Histogram and quantitative data of the pro-inflammatory cytokines TNF (A) and IL-1β (B) in infiltrating myeloid cells identified as CD45^{hi}CD11b^{+} via flow cytometry from the injury spinal cord at 3 d post-injury. SCI resulted in higher mean fluorescent intensity (MFI) of TNF and IL-1β in Ly6C^{hi} monocyte and Ly6G^{+} neutrophil infiltration in both WT and *Becn1*$^{+/-}$ mice. However, our data showed no differential change between the genotypes. n = 9 (SCI/WT) and 8 (SCI/*Becn1*$^{+/-}$) mice.

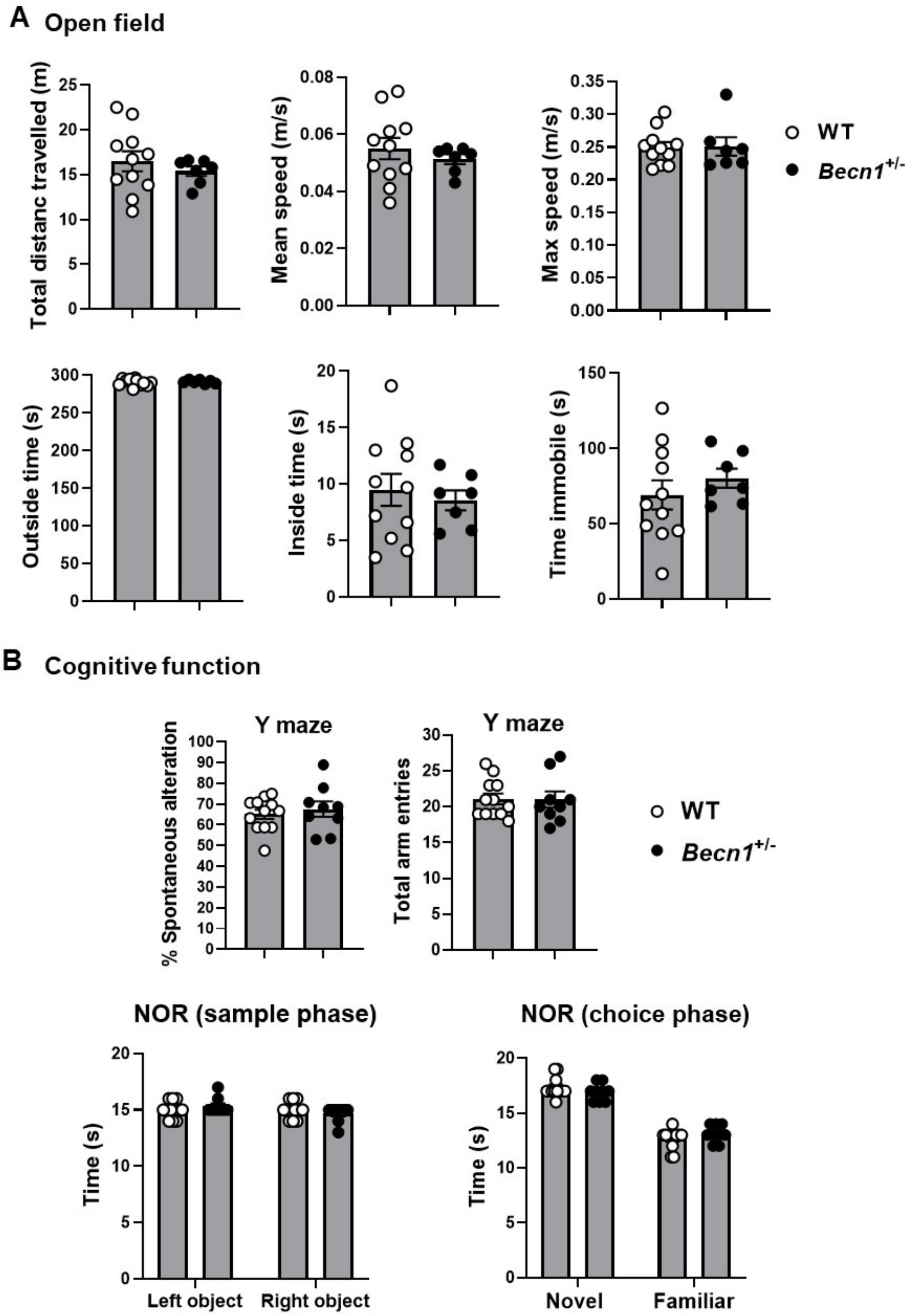

Figure S7. Autophagy deficiency had no effect on spontaneous motor and cognitive function in *Becn1*$^{+/-}$ mice. (**A**) Open field was used to assess spontaneous motor activity for WT and *Becn1*$^{+/-}$. No genotype differences were observed in the 6 parameters examined. n = 11 (WT) and 7 mice (*Becn1*$^{+/-}$). (**B**) Cognitive function examined with Y-maze and novel object recognition (NOR) tests. Neither percentage of spontaneous alteration nor exploration time during choice phase showed genotype differences. n = 12 (WT) and 9 mice (*Becn1*$^{+/-}$) Unpaired t-test.
